# Infrared Photodissociation Spectroscopy of Dinuclear Vanadium-Group Metal Carbonyl Complexes: Diatomic Synergistic Activation of Carbon Monoxide

**DOI:** 10.3390/molecules29122831

**Published:** 2024-06-14

**Authors:** Jin Hu, Xuefeng Wang

**Affiliations:** Shanghai Key Laboratory of Chemical Assessment and Sustainability, School of Chemical Science and Engineering, Tongji University, 1239 Siping Road, Shanghai 200092, China; hujin328@tongji.edu.cn

**Keywords:** infrared photodissociation spectroscopy, density functional calculation, vanadium group metal carbonyl complexes, CO activation

## Abstract

The geometric structure and bonding features of dinuclear vanadium-group transition metal carbonyl cation complexes in the form of VM(CO)*_n_*^+^ (*n* = 9–11, M = V, Nb, and Ta) are studied by infrared photodissociation spectroscopy in conjunction with density functional calculations. The homodinuclear V_2_(CO)_9_^+^ is characterized as a quartet structure with C_S_ symmetry, featuring two side-on bridging carbonyls and an end-on semi-bridging carbonyl. In contrast, for the heterodinuclear VNb(CO)_9_^+^ and VTa(CO)_9_^+^, a C_2V_ sextet isomer with a linear bridging carbonyl is determined to coexist with the lower-lying C_S_ structure analogous to V_2_(CO)_9_^+^. Bonding analyses manifest that the detected VM(CO)_9_^+^ complexes featuring an (OC)_6_M–V(CO)_3_ pattern can be regarded as the reaction products of two stable metal carbonyl fragments, and indicate the presence of the M–V *d-d* covalent interaction in the C_S_ structure of VM(CO)_9_^+^. In addition, it is demonstrated that the significant activation of the bridging carbonyls in the VM(CO)_9_^+^ complexes is due in large part to the diatomic cooperation of M–V, where the strong oxophilicity of vanadium is crucial to facilitate its binding to the oxygen end of the carbonyl groups. The results offer important insight into the structure and bonding of dinuclear vanadium-containing transition metal carbonyl cluster cations and provide inspiration for the design of active vanadium-based diatomic catalysts.

## 1. Introduction

Transition metals, especially noble metals, are ubiquitous in catalysis and material chemistry and remain the indispensable catalysts in numerous chemical reactions. The scarcity, high cost, toxicity as well as concerns about the adverse environmental impacts from the extraction, purification, utilization and reclamation of transition metals have inspired efforts to find ways to reduce the use of transition metals. Single atom catalysts (SACs) are being pursued as attractive solutions to the aforementioned problem by virtue of their maximized metal atom utilization, well-defined active sites and exceptional selectivity compared with traditional bulk material and supported nano-particles [1,2,3]. However, despite a true boost in catalytic performance and the feasibility of further improving their intrinsic activity by careful tuning of their coordination and electronic configurations, the poor structural and compositional complexity of SACs renders them inadequate in the face of complicated reactions. In addition, the lack of diverse synergistic interactions in SAC materials with only a single type of active site presents an insurmountable obstacle to regulating activity and selectivity over a wide range [4]. This calls for the introduction of additional metal atoms in the vicinity of single active sites of SACs to form multi-atom cluster catalysts [5,6], typically such as dual atom catalysts (DACs), which allow for the inheritance of high atomic dispersity, high atomic utilization, high activity and selectivity of SACs, and the break of the intrinsic limitations as well. Studies of DACs to date have revealed opportunities in a broad variety of applications [7,8,9], but the molecular-level understanding of this diatomic synergistic effect remains elusive due to the herculean challenges to exploring the structure and properties of actual active sites fraught with complicated ambient perturbations through common characterization methods. Thus, a combination of spectroscopic methods and theoretical calculations to determine the interaction between diatomic metal clusters and small molecules is particularly meaningful.

Infrared photodissociation spectroscopy (IRPD) has proven to be a powerful tool allowing for structural identification of mass-selected charged clusters in the gas phase [10,11,12], as well as an understanding of bonding interactions between core atoms in conjunction with density functional calculations [13,14,15,16,17,18,19]. In addition, carbon monoxide (CO) acts as an ideal probe for detecting metal–ligand interactions owing to its strong infrared absorption and simple vibration pattern. Miscellaneous cationic and anionic carbonyl complexes of transition metals have been studied by combining infrared photodissociation spectroscopy with high-precision quantum computation over the past few decades [10,11,12]. The metal-carbonyl bonding is well-described as a combination of CO→M *σ*-donation and M→CO *π*-backdonation by the Dewar–Chatt–Duncanson (DCD) model [20,21]. The earlier transition metals are demonstrated to exhibit a stronger ability to activate CO due to their relatively smaller electron detachment energies, which is conducive to the π-backbonding. Zhou and coworkers systematically explored the structures and bonding of homodinuclear first-row transition metal carbonyl complex cations and found a predilection towards formation of (OC)*_x_*M–M(CO)*_y_* structure with terminally bound carbonyls for the saturated complexes of late transition metals such as Fe_2_(CO)_9_^+^ [22], Fe_2_(CO)_8_^−^ [23], Ni_2_(CO)_8_^+^ [24] and Cu_2_(CO)_6_^+^ [25]. Similar structural and bonding features have also been shown to prevail in heterodinuclear late transition metal carbonyl complexes, including CuFe(CO)_7_^−^ [26], FeZn(CO)_5_^+^ [27], CoZn(CO)_7_^+^ [27], FeM(CO)_8_^+^ (M = Co, Ni, Cu) [28] and MCu(CO)_7_^+^ (M = Co, Ni) [28]. The carbonyl groups in these complexes are separately tethered to the two metal centers with a small shift of the infrared frequency relative to the free CO, signaling the poor ability of two late-transition metal atoms to synergistically activate CO. Moreover, these complexes exhibit a preference for asymmetric structures, so as to give priority to ensuring that one of the metal centers meets the favorable 18-electron rule. In marked contrast, bridging CO ligands are presented in the homodinuclear carbonyl complexes of early transition metals. The Cr_2_(CO)_9_^+^ cation was characterized to have the (OC)_5_Cr–C–O–Cr(CO)_3_ configuration with a linear bridged carbonyl group bonded to one chromium site through its carbon atom and to the other chromium site through its oxygen atom [29]. The Ti_2_(CO)_9_^+^ cation was determined to be a doublet C_S_ structure with two side-on bridging carbonyls and one semi-bridging carbonyl [30]. The carbonyl group serving as a four-electron donor in these complexes is simultaneously activated by two metal atoms, thus featuring an appreciably elongated C–O distance and a significant redshift in the stretching frequency.

Atomically dispersed vanadium-related catalysts are ubiquitous in various reactions including the oxidations of alkanes, alkenes, arenes, alcohols, as well as the activation and cleavage of C–C and C–O bonds [31]. Atomically precise small size clusters in the gas phase are ideal models for studying the cluster chemical bonding and the cluster–ligand interactions. Recently, clusters related to vanadium-group metals have also received special attention [32,33,34]. However, spectroscopic investigations involving multinuclear vanadium-containing complexes with metal–metal synergistic interactions are rarely available, since the formation and stabilization of pure vanadium-group all-metallic clusters in the gas phase is experimentally challenging due to the intense propensity of vanadium-group metals to form oxides [35,36,37,38,39].

In the present work, homo- and heterodinuclear vanadium-containing carbonyl complex cations in the form of VM(CO)*_n_*^+^ (M = V, Nb, and Ta; *n* = 9–11) are produced in the gas phase and studied by infrared photodissociation spectroscopy. The geometric structures of mass-selected complexes are determined by comparisons of their experimental infrared photodissociation spectra and simulated IR spectra from density functional calculations.

## 2. Results

### 2.1. Mass Spectra

**Figure 1:**
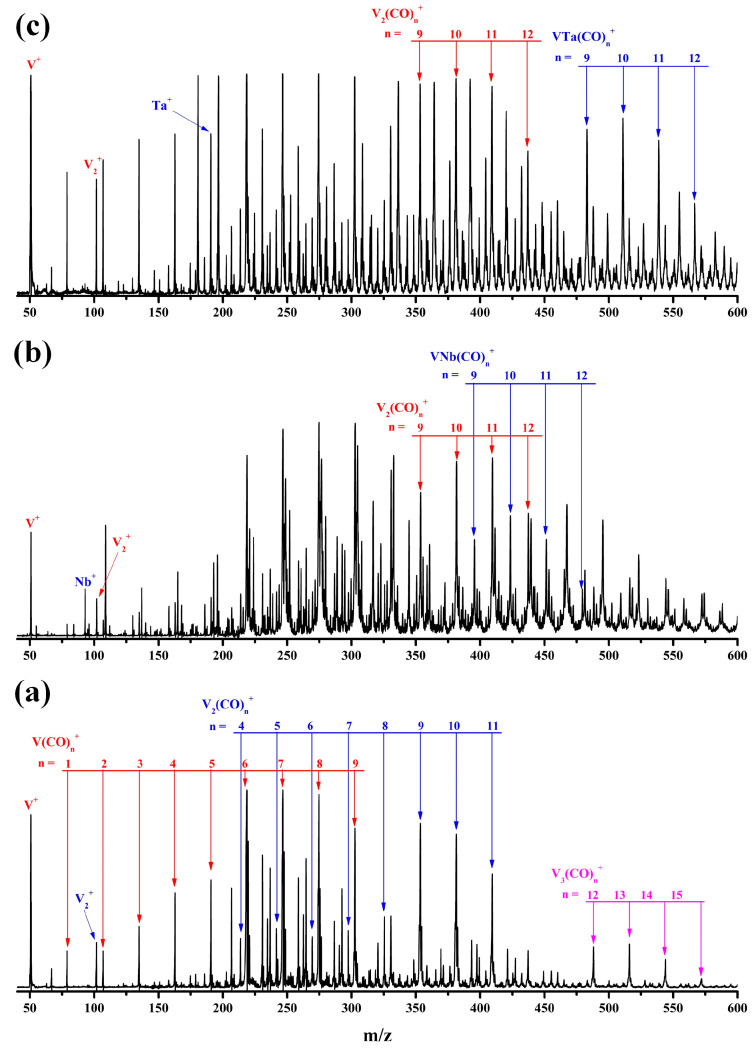
The representative mass spectrum of the cation complexes in the *m*/*z* range of 45–600 produced by pulsed laser ablation of a pure vanadium target (**a**), the mixed vanadium–niobium target (**b**), and vanadium–tantalum target (**c**) in an expansion of 10% CO seeded in helium at a background pressure of 1.2 MPa.

The representative mass spectrum of cations generated by the pulsed laser ablation of the pure bulk vanadium target in the expansion of a CO/He mixture is shown in Figure 1a. The product progressions are completely composed of the homonuclear carbonyl complexes V*_m_*(CO)*_n_*^+^ with m = 1–3. For the mixed vanadium–niobium and vanadium–tantalum targets, aside from the exclusively vanadium-containing mono/multinuclear carbonyl complexes, the heteronuclear bimetallic carbonyl complexes VNb(CO)*_n_*^+^ and VTa(CO)*_n_*^+^ were observed (Figure 1b,c). The chemical formulas of each compound were determined by combining their mass-to-charge ratios with the most probable carbonyl coordination number at the metal centers.

Duncan and coworkers reported that for a single vanadium cation, the 16-electron V(CO)_6_^+^ is saturated and the formation of strongly bonded 18-electron V(CO)_7_^+^ is prohibited in the pulsed laser sputtering source [40,41]. As displayed in Figure 1a, the V(CO)_6_^+^ cation shows a sharp increase in the mass intensity relative to V(CO)_1–5_^+^, followed by a smooth decrease in the intensity of V(CO)_7–9_^+^, indicating that the current experimental conditions are biased in favor of the formation of saturated coordination compounds. Accordingly, the V_2_(CO)_9_^+^ and V_3_(CO)_12_^+^ cations with the highest abundance in their corresponding product sequences are expected to be the saturated homogeneous dinuclear and trinuclear vanadium carbonyl complexes, respectively. It is worth noting that from V(CO)_6_^+^ over V_2_(CO)_9_^+^ to V_3_(CO)_12_^+^, one V(CO)_3_ unit is progressively added, to some extent suggesting the possible presence of a certain rule in the growth process of multinuclear vanadium carbonyl complexes. Except for the heterodinuclear VNb(CO)_10,11_^+^ and VTa(CO)_10,11_^+^ cations, the similar mass intensity indicates that their saturated coordination numbers cannot be determined unilaterally by the mass spectrum.

**Figure 2:**
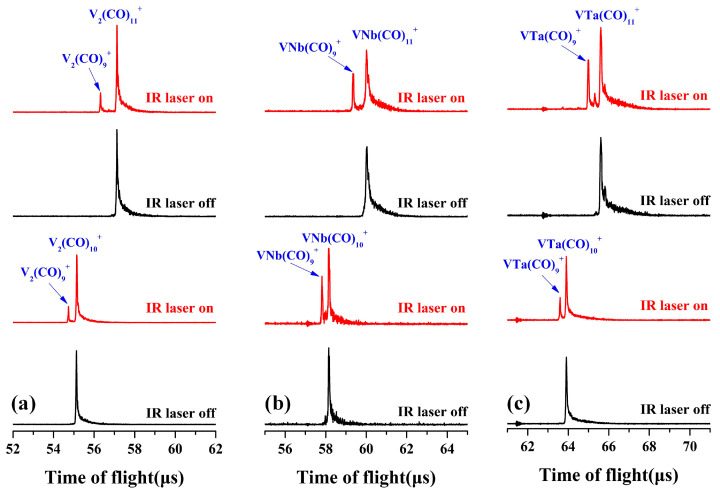
The laser-induced photofragmentation mass spectra of mass-selected V_2_(CO)_10,11_^+^ (**a**), VNb(CO)_10,11_^+^ (**b**), and VTa(CO)_10,11_^+^ (**c**). The laser used in (**a**) is set at 2130 cm^−1^ with an energy of 1.0 mJ/pulse, and the laser used in (**b**,**c**) is set at 2150 cm^−1^ with an energy of 1.2 mJ/pulse.

The VM(CO)_9_^+^ (M = V, Nb, and Ta) complexes were mass-selected to interact with tunable infrared laser in the carbonyl stretching frequency region. However, no dissociation was observed, indicating that these nonacarbonyl compounds are strongly bonded substances. Further, the decacarbonyl and undecacarbonyl complexes VM(CO)_10,11_^+^ (M = V, Nb, and Ta) were mass-isolated and exposed to infrared laser radiation, and obvious laser-induced fragmentation occurred (Figure 2). The VM(CO)_10_^+^ cations rapidly dissociate through the immediate removal of a CO ligand at a laser energy of 1.0–1.2 mJ/pulse, resulting in the decomposition of about 20–30% of the parent ions into the nonacarbonyl species. The direct shedding of double CO ligands from the parent cation VM(CO)_11_^+^ with a higher dissociation at the same frequency of laser radiation was observed, indicating that the nonacarbonyl VM(CO)_9_^+^ complexes are probably fully coordinated and the outermost two CO ligands are exceedingly weakly attached to the periphery of the transition metal saturated coordination sphere.

### 2.2. Infrared Photodissociation Spectra

Labeling techniques by tagging a weakly bound ligand can help indirectly obtain the infrared photodissociation spectra of strongly bonded substances. In this regard, the VM(CO)_10,11_^+^ cations generated under experimental conditions conducive to the formation of oversaturated complexes were selected for photodissociation. The infrared photodissociation spectra of V_2_(CO)_10,11_^+^ present totally identical number and location of adsorption peaks, despite slight shifts of some bands within 5 cm^−1^ as well as insignificant differences in intensity (Figure S1). Similarly, the resulting photodissociation spectra of VNb(CO)_11_^+^ and VTa(CO)_11_^+^ from monitoring the simultaneous volatilization of two labeling CO molecules are nearly identical to that of VNb(CO)_10_^+^ and VTa(CO)_10_^+^, respectively (Figures S2 and S3). The spectroscopic observations well justify the rationality of the presumption that VM(CO)_9_^+^ are saturated complexes.

**Figure 3:**
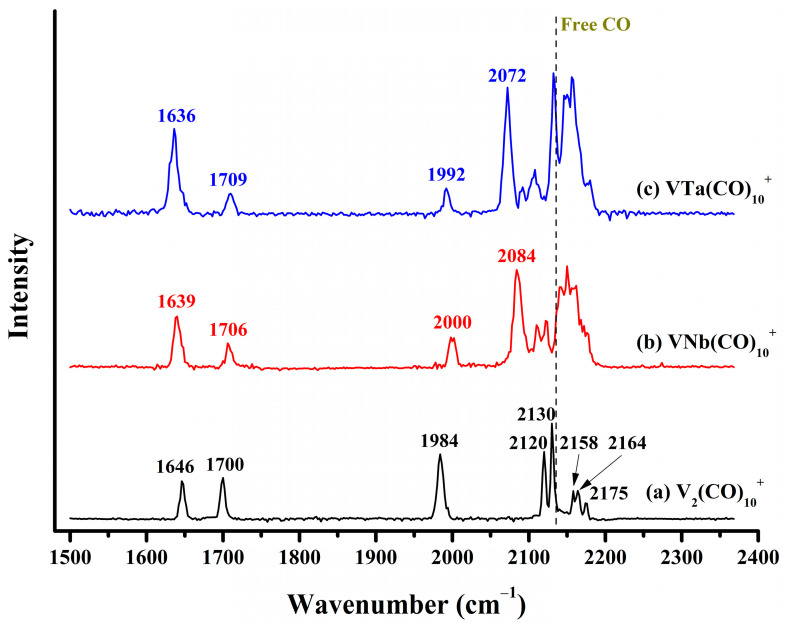
The experimental infrared photodissociation spectra of (**a**) V_2_(CO)_10_^+^, (**b**) VNb(CO)_10_^+^ and (**c**) VTa(CO)_10_^+^ via elimination of the outermost physically bound CO in the carbonyl stretching frequency region, leading to the formation of VM(CO)_9_^+^ (M = V, Nb, and Ta). The vertical dashed line indicates the C–O vibration frequency of free CO at 2143 cm^−1^.

The infrared photodissociation spectrum of the VM(CO)_10_^+^ (M = V, Nb, Ta) cations are collectively displayed in Figure 3. For V_2_(CO)_10_^+^, eight well-resolved absorption bands centered at 1646, 1700, 1984, 2120, 2130, 2158, 2164 and 2175 cm^−1^ were detected. The observation of two bands far below 2000 cm^−1^ and one band slightly below 2000 cm^−1^ indicates that this cation complex involves multiple bridge-bonded CO ligands in different forms. For VNb(CO)_10_^+^ and VTa(CO)_10_^+^, the three low-frequency absorption bands associated with bridged carbonyls were also detected at roughly similar frequency regions, suggesting some similarity between them and the ground state structure of V_2_(CO)_10_^+^. In contrast, in the frequency region between 2050 and 2200 cm^−1^, VNb(CO)_10_^+^ and VTa(CO)_10_^+^ exhibit more absorption bands compared to V_2_(CO)_10_^+^. On the whole, the profiles of infrared photodissociation spectra of VNb(CO)_10_^+^ and VTa(CO)_10_^+^ are highly consistent but slightly different from that of V_2_(CO)_10_^+^. One possible scenario is that in addition to the ground state structure shared with V_2_(CO)_10_^+^, VNb(CO)_10_^+^ and VTa(CO)_10_^+^ have other isomers that are allowed under our experimental conditions. Anyway, the high resemblance in the photodissociation spectra of the VM(CO)_10_^+^ cations implies similar geometric parameters and bonding situations of corresponding saturated coordination VM(CO)_9_^+^ complexes.

### 2.3. Structure of the VM(CO)_9_^+^ Cations

**Figure 4:**
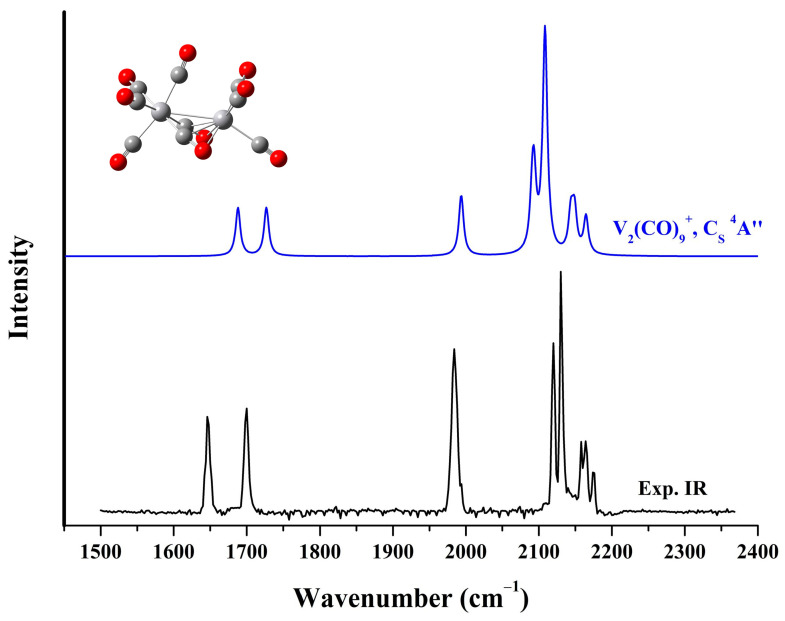
The experimental infrared photodissociation spectrum of V_2_(CO)_10_^+^ and the simulated vibrational spectrum of the saturated V_2_(CO)_9_^+^ cation complexes in the carbonyl stretching frequency region.

Quantum chemical calculations based on density functional theory were conducted to support the assignments of the vibrational spectra of the observed species and to examine the geometric and electronic structure of the carbonyl complexes. The calculated eight lowest-lying structural isomers of V_2_(CO)_9_^+^ at the B3LYP, BLYP, PBE, and TPSS level of theory are presented in Figure S4. And the comparison of the experimental photodissociation spectrum of the solvated V_2_(CO)_10_^+^ with the simulated spectra of these stable candidates derived from the B3LYP calculations is shown in Figure S5. The global minimum (a) has a ^4^E electronic state and C_3V_ symmetry with three equivalent end-on semi-bridging carbonyl groups and six terminally bonded carbonyl groups at two vanadium centers. The second structure (b) has C_S_ symmetry involving two side-on bridging CO ligands and one end-on semi-bridging CO ligand. Structure (c) is also a C_S_-symmetry structure, but has two end-on semi-bridging carbonyls and one side-on bridging carbonyl. In contrast to these three lowest-lying structures, all of which are quartet structures, the fourth structure (d) is a sextet structure that appears as a (OC)_5_V–C–O–V(CO)_3_ configuration with a linear-bridge carbonyl group. The subsequent isomers (e), (f), (g) and (h) are all characterized by one or more similar quasi-linear bridging carbonyls. The C_S_ isomer (b) provides a simulated infrared spectrum that well agrees with the experimental photodissociation spectrum (Figure 4). In this structure, the two side-on bridging carbonyls are equivalent, with an appreciably elongated C–O distance relative to the end-on semi-bridging carbonyl and the other terminal CO ligands. The theoretical frequencies for the C–O stretching modes of the VM(CO)_9_^+^ cations, along with the experimental values are shown in Table 1. The calculated values are derived from the scaled harmonic frequencies from calculations at the B3LYP/def2-TZVP level with a factor of 0.968, which comes from the ratio of the fundamental frequency of free CO (2143 cm^−1^) to the calculated value (2214 cm^−1^). For V_2_(CO)_9_^+^, the very low CO stretching frequencies at 1646 and 1700 cm^−1^ are attributed to the antisymmetric and symmetric stretching vibrations of the two four-electron donor side-on bridging carbonyl groups, respectively. The single band centered at 1984 cm^−1^ is due to the stretching vibration of the end-on semi-bridging CO ligand. The 2120 and 2130 cm^−1^ bands originate from the coupled stretching vibrations of the three end-on carbonyl groups on the left-hand vanadium atom. In contrast, the blue-shifted bands at 2158, 2164 and 2175 cm^−1^ result from the three carbonyl groups terminally bonded on the right-hand vanadium atom.

As a further supplement, geometry optimizations were performed on various possible structures for V_2_(CO)_10_^+^. As shown in Figure S6, the most stable structure (a) at the B3LYP level can be viewed as the further reaction product of the fourth sextet V_2_(CO)_9_^+^ with a CO ligand, which maintains the linear (OC)_5_V–C–O–V(CO)_4_ structure. The sub-stable C_2V_-symmetry structure (b) can also be seen as the formation of the isomer (h) of V_2_(CO)_9_^+^ with an additional CO molecule, retaining the characteristic two quasi-linear bridging carbonyl groups as four-electron donors. The other isomers lie at least 4 kcal/mol higher in energy relative to the global minimum, all of which exhibit bridging carbonyls with specific patterns. As shown in Figure S7, none of the simulated infrared spectra of the eight lowest-lying isomers of V_2_(CO)_10_^+^ can match the experimental spectrum of V_2_(CO)_10_^+^, confirming that the V_2_(CO)_10_^+^ cation observed in the experiments is a loose adduct formed from the saturated coordination product V_2_(CO)_9_^+^ with a weakly adsorbed CO molecule. A comparison of the experimental photodissociation spectrum of V_2_(CO)_10_^+^ with the calculated infrared spectrum of the saturated V_2_(CO)_9_^+^ cation as well as that of the CO-tagged V_2_(CO)_10_^+^ is presented in Figure S8. The disturbance of the outlying CO ligand to the geometrical parameters and chemical bonding of the fully coordinated V_2_(CO)_9_^+^ core is negligible, except for slight shifts of the main absorption bands.

**Figure 5:**
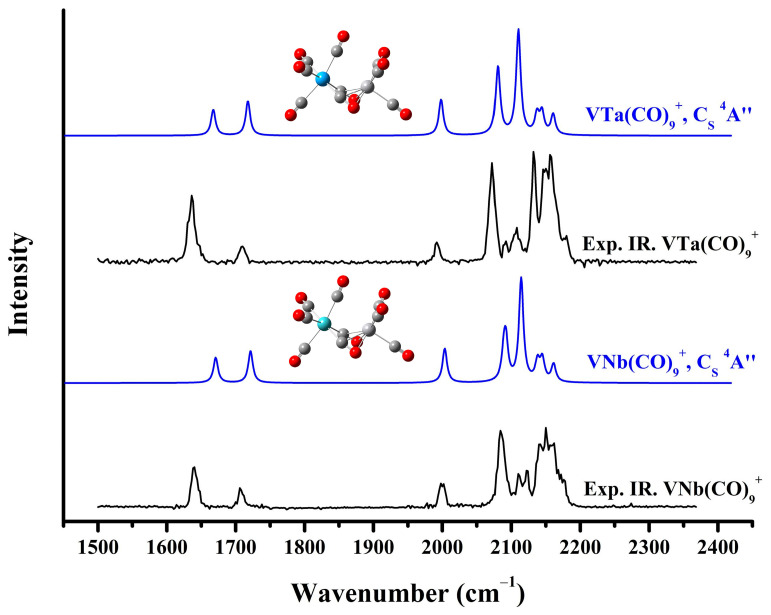
The experimental infrared photodissociation spectra of VNb(CO)_10_^+^ and VTa(CO)_10_^+^ and the simulated vibrational spectra of the saturated VNb(CO)_9_^+^ and VTa(CO)_9_^+^ cation complexes in the carbonyl stretching frequency region.

For the VNb(CO)_9_^+^ cation, eight low-lying structures and comparisons of their simulated spectra with the experimental infrared photodissociation spectrum of VNb(CO)_10_^+^ are displayed in Figure S9 and Figure S10, respectively. The lowest-lying structures (a), (b) and (c) share similar geometric parameters and electronic states with the three most stable isomers of V_2_(CO)_9_^+^, respectively. In fact, the geometric features of structures (d)–(g) are also consistent with several low-energy isomers of V_2_(CO)_9_^+^, despite a varying order of energies at the computation level with different functionals. Three low-frequency absorption bands below 2000 cm^−1^ firmly confirm the presence of structure (b) at the B3LYP level, which has the same C_S_ symmetry and ^4^A” electron state as the structure (b) of V_2_(CO)_9_^+^. Unfortunately, the intensity of the two absorption bands near 1700 cm^−1^ predicted by theoretical calculation is roughly the same, while the experimental results clearly show that the absorption band centered at 1639 cm^−1^ is much more intense than the band at 1706 cm^−1^. In addition, the absorption bands attributed to terminal carbonyl groups between 2050 and 2200 cm^−1^ of structure (b) cannot match the experimental results very well. We noted that the throng of absorption bands of terminally bonded carbonyl groups of the sextet structure (e) provides a reasonable agreement with the experimental spectrum. However, according to the thermodynamic energies, the sextet isomer of VNb(CO)_9_^+^ stands more than 23 kcal/mol higher relative to the calculated C_S_-symmetry structure (b) at the PBE level with def2-TZVP basis set, as shown in Figure S9C. Such a significant energy difference suggests that the coexistence of these two structures is unlikely. Thus, as shown in Figure 5, the experimentally observed VNb(CO)_9_^+^ is attributed to the C_S_-symmetry structure. Figure S11 displays the eight lowest-lying isomers of the strongly bonded VNb(CO)_10_^+^ cation complex. The structures with thermodynamic advantages have a preference for the electronic states of quartet. As shown in Figure S12, none of the simulated spectra of isomers match the experimental spectra of VNb(CO)_10_^+^, showing no evidence for the formation of strongly bonded VNb(CO)_10_^+^ under our experimental conditions. Note that although the photofragmentation mass spectra of VM(CO)_11_^+^ (Figure 2) and their spectra show no evidence for the strongly bonded VM(CO)_10_^+^, their much lower energies relative to the solvated CO-tagged VM(CO)_10_^+^ still suggest that they may be available under altered experimental conditions.

**Figure 6:**
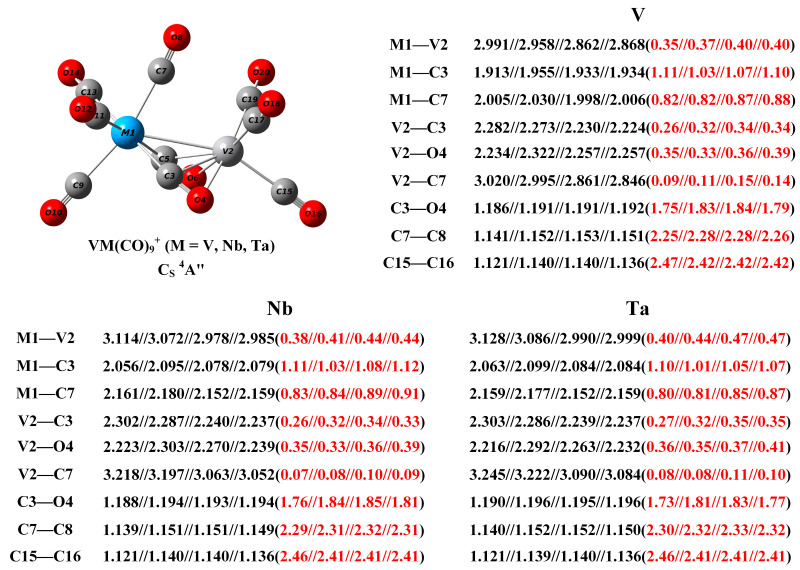
The geometric structure of the VM(CO)_9_^+^ (M = V, Nb, and Ta) cations, as well as the calculated bond distance (in Å, black font) and Mayer bond order (red font in parentheses) of selected bonds at the B3LYP, BLYP, PBE, and TPSS level with the def2-TZVP basis set.

The calculated lowest-lying structures of the strongly bonded VTa(CO)_9_^+^ and the comparison of their simulated vibrational spectra with the experimental infrared photodissociation spectrum of VTa(CO)_10_^+^ are presented in Figure S13 and Figure S14, respectively. As in the case of VNb(CO)_9_^+^, the VTa(CO)_9_^+^ cation generated in the experiment was determined to be the C_S_-symmetry quartet structure (Figure 5). Similarly, no strongly bonded VTa(CO)10+ complex provides a calculated spectrum matching the experimental spectrum (Figures S15 and S16), showing no evidence for its presence in this experiment. To sum up, based on experimental results and theoretical calculations, the structure of VM(CO)_9_^+^ (M = V, Nb, and Ta) complexes with C_S_ symmetry and a ^4^A” electronic state is indisputably established (Figure 6). According to the calculated frequencies in Table 1, for VNb(CO)_9_^+^ and VTa(CO)_9_^+^, the two lowest-frequency bands detected in the experiment are mainly attributed to the antisymmetric and symmetric stretching vibration of the two side-on bridging carbonyls, respectively, and the single band close to 2000 cm^−1^ is due to the stretching vibration of the end-on semi-bridging carbonyl. Notably, VM(CO)_9_^+^ are all quartet complexes, while previous studies have shown that bare V_2_^+^ also has a quartet ground state [42]. This may mean that the CO ligands only coordinate into the unoccupied orbitals of V_2_^+^ over the course of the formation of saturated carbonyl complexes, without affecting the configuration of its unpaired electrons.

## 3. Discussion

The calculated bond distance and Mayer bond index for some bonds of interest, along with the geometrical structures of the VM(CO)_9_^+^ cations are shown in Figure 6. The V–V distance is predicted to be 2.868 Å with a Mayer bond index of 0.40 at the TPSS level, indicating the presence of a half-bond. The V1–C3 distance is estimated to be 1.934 Å, which is much shorter than the V1–C7 bond (2.006 Å), suggesting that the covalent interaction between the side-on bridging carbonyl and the V1 center is significantly enhanced compared to that of the end-on semi-bridging carbonyl and other terminal carbonyls. The C–O distance of the side-on bridging carbonyl is appreciably elongated to 1.192 Å, roughly 5% longer than the bond length of free CO (1.135 Å). And its Mayer bond index is calculated to be 1.79, unequivocally proving that this carbonyl group is highly activated and closer to a C=O double bond. The calculated Mayer bond orders of the V2–C3 bond (0.34) and V2–O4 bond (0.39) demonstrate that there exist strong chemical bonding interactions between the right-hand vanadium atom and the side-on bridging carbonyl. From V over Nb to Ta, the M1–V2 bond is much elongated, but the calculated bond index is monotonically increasing. The longer bond distance is attributed to the increasing radius of vanadium-group metals from top to bottom, while the increased bond order indicates an enhanced metal–metal interaction. The modest decrease in the bond index of the Ta1–C3 and Ta1–C7 bonds relative to the Nb1-C3 and Nb1-C7 bonds can be rationalized by the contraction of *s* and *p* orbitals due to the relativistic effect of heavy atoms [43]. Thus, the increase in the M1–V2 bond index from V over Nb to Ta suggests that this bond is mainly related to the *d* orbital in the metal center.

**Figure 7:**
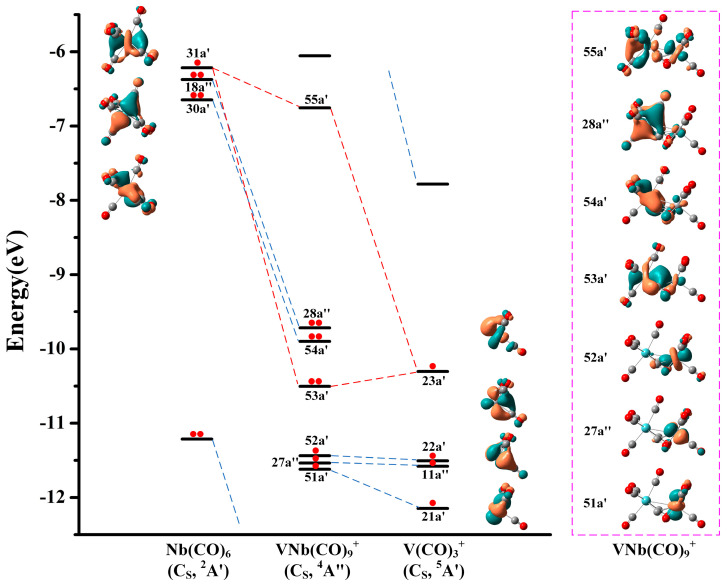
Bonding scheme for the orbital interactions between the doublet Nb(CO)_6_ fragment and the quintet V(CO)_3_^+^ fragment, as well as the canonical Kohn–Sham molecular orbitals of the C_S_ structure of the quartet VNb(CO)_9_^+^ at the TPSS/def2-TZVP level. The electrons in the corresponding orbitals are marked with red dots. All the molecular orbitals are plotted with isosurfaces = 0.05 a.u.

To gain deeper insight into the electronic structures and chemical bonding of the VM(CO)_9_^+^ cations, the natural population analysis was conducted. As displayed in Table S1, the M1 center of the quartet VM(CO)_9_^+^ is substantially negative, while the V2 center is relatively positive. More importantly, the spin population on the V2 center exceeds 2.50 e, indicating that all unpaired electrons are highly localized. Therefore, the experimentally determined VM(CO)_9_^+^ structures can be regarded as reaction products of a neutral M(CO)_6_ fragment with a cationic high-spin V(CO)_3_^+^ fragment. Note that Duncan and coworkers have reported the infrared photodissociation spectrum of the V(CO)_3_^+^ cation by argon labeling technique, and determined its T-shaped geometry and quintet electronic state in combination with density functional calculations [41]. Moreover, the evident abundance of V(CO)_3_^+^ in the mass spectra (Figure 1) also supports the assignment that it functions as a raw material for the generation of VM(CO)_9_^+^. The correlation diagram for the orbital interactions along with the frontier canonical Kohn–Sham valence molecular orbitals of VNb(CO)_9_^+^ is shown in Figure 7. The HOMO (28a″) and HOMO-1 (54a′) of VNb(CO)_9_^+^ principally originate from the 18a″ and 30a′ orbitals of the Nb(CO)_6_ fragment, respectively, representing two non-bonding orbitals. The Nb(CO)_6_ fragment possesses an electronic structure of 4*d*^9^5*s*^2^5*p*^6^ with a half-filled *d*-type orbital (SOMO, 31a′). This singly occupied orbital interacts with the SOMO (23a′) of the quintet V(CO)_3_^+^ fragment in a covalent manner, forming the bonding orbital 53a′ and the antibonding orbital 55a′ of VNb(CO)_9_^+^. Notably, the three singly occupied orbitals of VNb(CO)_9_^+^ (51a′, 27a″ and 52a′) energetically stand much lower than the doubly occupied 53a′, 54a′ and 28a″ orbitals, which seems unusual because unpaired electrons usually behave actively and therefore reside in the outermost orbitals. It can be rationalized by the description that these orbitals are derived from the 21a′, 11a″ and 22a′ orbitals of the V(CO)_3_^+^ fragment. The desire to minimize the thermodynamic energy of the product from the fragment reaction drives these single-occupied orbitals to remain in the inner orbitals. Therefore, the V–Nb interactions in the quartet VNb(CO)_9_^+^ cation can be described as a single electron-sharing bond, which renders the niobium center the favored 18-electron configuration. This bonding scheme is also established for the C_S_ structures of V_2_(CO)_9_^+^ and VTa(CO)_9_^+^.

**Figure 8:**
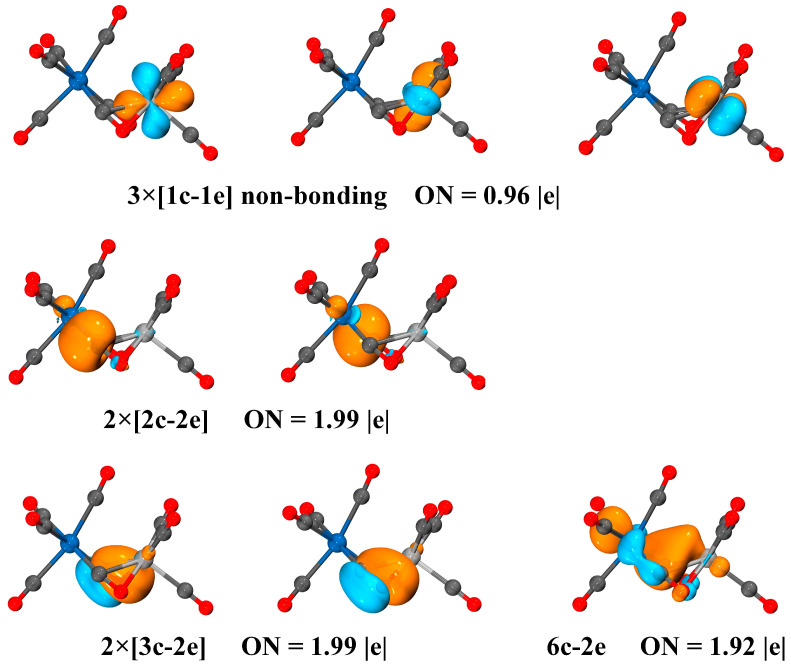
Localized nc-ne bonds and occupation numbers in the quartet VNb(CO)_9_^+^ obtained by AdNDP analyses at the TPSS/def2-TZVP level. All the molecular orbitals are plotted with isosurfaces = 0.05 a.u.

According to the AdNDP analysis shown in Figure 8, the bonding interaction related to the two side-on bridging carbonyls in VNb(CO)_9_^+^ consists of two two-center-two-electron (2c-2e) Nb–C bonds with occupation numbers of 1.99 |e|, two 3c-2e bonds with an occupation number of 1.99 |e|, and one 6c-2e bond with the occupation number of 1.92 |e|. The two 2c-2e Nb–C bonds represent the σ donation of the side-on bridging carbonyls to the Nb center. The two 3c-2e bonds illustrate the formation of π donation of the side-on bridging carbonyls to the V center. Accordingly, the two side-on bridging carbonyl groups act as both the classical σ donor and the less common π donor, essentially serving as four-electron donors. The 6c-2e bond depicts the interaction between the two metal centers, which principally consists of the *d_xy_*, *d_x2-y2_* and *d_z2_* orbitals of V/Nb and partly contains the contribution of the 2π^*^ orbitals of the side-on bridging carbonyls (Table S2). This *d-d* bonding is consistent with the results of the fragment interaction analysis.

## 4. Methods

The homodinuclear vanadium carbonyl cluster cations were produced by the pulsed laser ablation of a pure vanadium target. And the heteronuclear vanadium–niobium, vanadium–tantalum carbonyl cluster cations were produced by the pulsed laser ablation of composite targets, which were prepared via direct compression of the mixture of vanadium powder and niobium/tantalum powder with a molar ratio of 1:3. The 1064 nm fundamental of a Nd: YAG laser (Continuum, Minilite II, 10 Hz repetition rate and 6 ns pulse width) was introduced to the rotating metal target by the optical lens, leading to the formation of a searing metallic plasma. Various carbonyl complexes were produced from the reactions of the hot plasma with 10% CO seeded in helium using a pulsed valve (General Valve, Series 9) at a background pressure of 1.0–1.5 MPa. The prepared products were cooled by supersonic adiabatic expansion and were skimmed to be analyzed using a Wiley–McLaren time-of-flight mass spectrometer. The ions of interest were mass-selected by a mass gate and forced to interact with a tunable infrared laser beam provided by an OPO/OPA system (Laser Vision) pumped by a Nd:YAG laser (Continuum, Surelite EX). Under the laser radiation with a frequency consistent with the vibration of cluster complex, the energy of the infrared photon is absorbed by the complex. Over the course of energy relaxation, the weakest links in the molecule may be broken. The bond energy of a typical chemical bond is generally higher than the energy of a single infrared photon, so it is difficult to directly measure the infrared photodissociation spectrum of strongly bonded species by using the infrared laser from the OPO/OPA optical system. The spectrum of the complex of interest can be obtained indirectly by the labelling technique via weakly bound messengers. Therefore, the solvated loose complex were mass-isolated for photodissociation in this experiment.

The dissociated fragments and parent cations were reaccelerated and mass-analyzed by the second tandem TOF mass spectrometer. The infrared photodissociation spectra were obtained by monitoring the yield of the fragment cations as a function of the infrared laser wavelength with a scan step of 2 cm^−1^. In this experiment, the used IR laser beam has an energy of 0.7–1.3 mJ/pulse.

First principle density functional theory (DFT) calculations were carried out using the Gaussian 09 computational package [44]. The hybrid B3LYP functional [45,46] is the most extensively used density functional for structural optimization and harmonic vibration frequency analysis, which has been demonstrated to be able to provide reliable predictions on the structures and vibrational frequencies of transition metal-containing compounds [47,48,49]. Therefore, the preliminary structure search for each carbonyl complex VM(CO)*_n_*^+^ was conducted using the def2-SVP basis set at the B3LYP level [50,51], which started from a plethora of initial structures generated by randomly putting the CO ligands around the exposed VM^+^ core. A large number of presupposed structures containing one/two/three bridging carbonyls sandwiched between the two metal centers in various binding patterns were considered as well [12]. The preliminary structure optimization yielded considerable candidates, in which the three lowest spin states, namely the doublet, quartet and sextet of each candidate isomer were considered. To the best of our knowledge, the B3LYP functional is capable of providing accurate vibrational spectra of carbonyl complexes involving transition metals [14,16,18,19], but is much inferior to the non-hybrid functionals in terms of energy calculation and bond length prediction [52,53,54]. Therefore, the resulting low-lying candidates from the preliminary structure search were further re-optimized at the B3LYP, BLYP, PBE, and TPSS level in conjunction with the def2-TZVP basis set to obtain more accurate energetical results and structural parameters [45,50,51,55,56,57,58]. We performed a calculation for the bond dissociation energy of the bare V_2_^+^ cation at the B3LYP, BLYP, PBE, and TPSS level with the def2-TZVP basis set. We found that the obtained results closest to the experimental value (3.140 eV ± 0.002 eV) are provided by the non-hybrid meta-GGA functional TPSS (3.970 eV) [42], as shown in Table S3. The non-hybrid BLYP and PBE calculations provide results that are more than 1 eV larger than the experimental value, while the hybrid functional B3LYP significantly underestimates the dissociation energy of V_2_^+^ (1.947 eV). Therefore, the thermodynamic energies derived from the recalculations at the non-hybrid TPSS/def2-TZVPP level are more reliable. After each optimization, the harmonic vibration frequency was carefully checked to confirm that a true minimum point was obtained, and the zero point vibration energy (ZPVE) was derived. The relative energies of various isomers were calculated based on the electron energies of related substances corrected by ZPVE. We have calculated the binding energy (in kcal/mol) of the weakly bound CO ligand in the solvated VM(CO)_10_^+^ at the B3LYP, BLYP, PBE, and TPSS level with the def2-TZVP basis set. As shown in Table S4, the binding energy predicted at all theoretical levels is within 2 kcal/mol, which is much smaller than the energy of a single infrared photon in our experiments (typically 1500–2300 cm^−1^, on the order of 7 kcal/mol), so the laser-induced CO dissociation from the solvated VM(CO)_10_^+^ is completely thermodynamically feasible. The computational spectra were scaled by a factor of 0.968 and widened by the Gaussian-type curve with a full width at half-maximum (FWHM) of 4 cm^−1^. The chemical bonding properties were analyzed by employing several different methods including Mayer bond order (MBO) [59], natural population analysis (NPA) [60], adaptive natural density partitioning (AdNDP) [61], orbital component analysis based on natural atomic orbital (NAO) [62] and orbital correlation based on charge decomposition analysis (CDA) [63]. All the bonding analyses were performed by using Multiwfn, which has proven to be a powerful wave function analysis software [64,65].

## 5. Conclusions

In summary, dinuclear vanadium-group transition metal carbonyl cation complexes in the form of VM(CO)*_n_*^+^ (M = V, Nb, and Ta) were produced in the gas phase via pulsed laser vaporization supersonic expansion and studied by infrared photodissociation spectroscopy in the carbonyl stretching frequency region. The geometric and electronic structures are assigned by comparison of the experimental spectra with simulated spectra derived from density functional calculations. The combination of laser-induced fragmentation mass spectra and infrared photodissociation spectra revealed that the observed VM(CO)_9_^+^ are saturated complexes, and there is no evidence to support the presence of the strongly bonded VM(CO)_10_^+^ under our experimental conditions. The dinuclear VM(CO)_9_^+^ cation can be irrefutably attributed to a quartet structure with C_S_ symmetry, featuring two highly activated side-on bridging carbonyls and one slightly activated end-on semi-bridging carbonyl. It is worth emphasizing that the C_S_ structure of VNb(CO)_9_^+^ and VTa(CO)_9_^+^ is characterized as the (OC)_6_M–V(CO)_3_ pattern, rather than the (OC)_6_V–M(CO)_3_ pattern, because the latter is much higher in energy. Accordingly, all the detected VM(CO)_9_^+^ complexes can be seen as the reaction products of two stable metal carbonyl fragments. Bonding analyses based on AdNDP and NAO methods along with the orbital correlation based on canonical fragment orbitals indicate the presence of the M–V *d-d* covalent interaction in VM(CO)_9_^+^, which renders the heavier metal center the favorable 18-electron configuration.

One of the striking results over the course of this work was the discovery of the vibrational bands below 1710 cm^−1^ of VM(CO)_9_^+^ cations, which represent highly activated CO ligands. DFT calculations manifest that the significant activation of the side-on bridging carbonyls in the VM(CO)_9_^+^ cations is due in large part to the diatomic cooperation of M–V, and the bridging carbonyls serve as both the classical σ donor and the less common π donor. The results offer important insight into the structure and bonding of dinuclear vanadium-containing transition metal carbonyl cluster cations and provide inspiration for the design of active vanadium-based diatomic catalysts.

## Figures and Tables

**Table 1:** Comparison of the calculated carbonyl stretching frequencies (in cm^−1^) of the VM(CO)_10_^+^ (M = V, Nb, and Ta) cations at B3LYP/def2-TZVP level of theory with the experimental values in the present work. IR intensities are listed in parentheses in km/mol, and all frequencies are scaled with a factor of 0.968.

Complex	Exptl.	Calc.	Mode ^a^
V_2_(CO)_10_^+^	1646	1688 (501)	C–O (side-on bridge) asym-str.
	1700	1727 (503)	C–O (side-on bridge) sym-str.
	1984	1994 (627)	C–O (end-on semi-bridge) str.
	2120	2091 (478), 2094 (628)	C–O (end-on at left-hand V) asym-str.
	2130	2108 (2332)	C–O (end-on at left-hand V) sym-str.
	2158	2143 (397)	C–O (end-on at right-hand V) asym-str.
	2164	2149 (428)	C–O (end-on at right-hand V) asym-str.
	2175	2164 (387)	C–O (end-on at right-hand V) sym-str.
VNb(CO)_10_^+^	1639	1671 (531)	C–O (side-on bridge) asym-str.
	1706	1721 (673)	C–O (side-on bridge) sym-str.
	2000	2004 (618)	C–O (end-on semi-bridge) str.
	2084, 2111, 2123, 2142, 2151, 2161, 2178	2089 (524), 2092 (773), 2114 (2161), 2137 (409), 2145 (458), 2162 (367)	C–O (terminal bound to V and Nb) asym-str./sym-str.
VTa(CO)_10_^+^	1636	1668 (589)	C–O (side-on bridge) asym-str.
	1709	1718 (662)	C–O (side-on bridge) sym-str.
	1992	1994 (605)	C–O (end-on semi-bridge) str.
	2072, 2092, 2107, 2133, 2149, 2158, 2180	2084 (553), 2090 (759), 2111 (2184), 2136 (421), 2142 (441), 2159 (338)	C–O (terminal bound to V and Nb) asym-str./sym-str.

^a^: The coupled carbonyl antisymmetric and symmetric stretching vibrations are marked as “asym-str.” and “sym-str.”, respectively, and the stretching vibration of a single carbonyl is indicated as “str.”.

## Data Availability

Data are contained within the article and Supplementary Materials.

## Supplementary Materials

The following supporting information can be downloaded at: https://www.mdpi.com/article/10.3390/molecules29122831/s1, Figure S1: The comparison of experimental infrared photodissociation spectra of the V_2_(CO)_10,11_^+^ cation complexes; Figure S2: The comparison of experimental infrared photodissociation spectra of the VNb(CO)_10,11_^+^ cation complexes; Figure S3: The comparison of experimental infrared photodissociation spectra of the VTa(CO)_10,11_^+^ cation complexes; Figure S4: The calculated lowest-lying structures of the strongly bonded V_2_(CO)_9_^+^ cation at the (A) B3LYP, (B) BLYP, (C) PBE, and (D) TPSS level with the def2-TZVP basis set; Figure S5: The experimental infrared photodissociation spectrum of V_2_(CO)_10_^+^ and the simulated vibrational spectra of the eight lowest-lying V_2_(CO)_9_^+^ cation complexes at the B3LYP/def2-TZVP level in the carbonyl stretching frequency region; Figure S6: The calculated lowest-lying structures of the strongly bonded V_2_(CO)_10_^+^ cation; Figure S7: The experimental infrared spectrum of V_2_(CO)_10_^+^ and the simulated vibrational spectra of the low-lying strongly bonded V_2_(CO)_10_^+^ cation complexes in the carbonyl stretching frequency region; Figure S8: Comparisons of the experimental infrared spectrum of V_2_(CO)_10_^+^ and the simulated vibrational spectra of the saturated V_2_(CO)_9_^+^ cation and the low-lying CO-tagged V_2_(CO)_10_^+^ complexes in the carbonyl stretching frequency region; Figure S9: The calculated lowest-lying structures of the strongly bonded VNb(CO)_9_^+^ cation at the (A) B3LYP, (B) BLYP, (C) PBE, and (D) TPSS level with the def2-TZVP basis set; Figure S10: The experimental infrared photodissociation spectrum of VNb(CO)_10_^+^ and the simulated vibrational spectra of the eight lowest-lying VNb(CO)_9_^+^ cation complexes at the B3LYP/def2-TZVP level in the carbonyl stretching frequency region; Figure S11: The calculated lowest-lying structures of the strongly bonded VNb(CO)_10_^+^ cation; Figure S12: The experimental infrared spectrum of VNb(CO)_10_^+^ and the simulated vibrational spectra of the low-lying strongly bonded VNb(CO)_10_^+^ cation complexes in the carbonyl stretching frequency region; Figure S13: The calculated lowest-lying structures of the strongly bonded VTa(CO)_9_^+^ cation at the (A) B3LYP, (B) BLYP, (C) PBE, and (D) TPSS level with the def2-TZVP basis set; Figure S14: The experimental infrared photodissociation spectrum of VTa(CO)_10_^+^ and the simulated vibrational spectra of the eight lowest-lying VTa(CO)_9_^+^ cation complexes at the B3LYP/def2-TZVP level in the carbonyl stretching frequency region; Figure S15: The calculated lowest-lying structures of the strongly bonded VTa(CO)_10_^+^ cation; Figure S16: The experimental infrared spectrum of VTa(CO)_10_^+^ and the simulated vibrational spectra of the low-lying strongly bonded VTa(CO)_10_^+^ cation complexes in the carbonyl stretching frequency region; Figure S17: The comparison between the experimental vibrational spectrum of the solvated V(CO)_7_^+^ cluster cation and the simulated spectra of saturated V(CO)_6_^+^ cation derived from calculations at (a) B3LYP, (b) BLYP, (c) PBE, and (d) TPSSh level in conjunction with the def2-TZVP basis set; Figure S18: The comparison between the experimental vibrational spectrum of the solvated V_2_(CO)_10_^+^ cluster cation and the simulated spectra of saturated V_2_(CO)_9_^+^ cation derived from calculations at (a) B3LYP, (b) BLYP, (c) PBE, and (d) TPSSh level in conjunction with the def2-TZVP basis set; Table S1: The calculated charge and spin population of VM(CO)_9_^+^ based on natural population analysis; Table S2: Orbital composition of the AdNDP orbitals of the quartet VNb(CO)_9_^+^ complex cations based on natural atomic orbital method; Table S3: The calculated energy (in a.u.) of quartet V_2_^+^ dimer, quintet V^+^ cation (3d^4^), and quartet V atom (4s^2^3d^3^), as well as the bond dissociation energy (BDE, in eV) at the B3LYP, BLYP, PBE, and TPSS level with the def2-TZVP basis set; Table S4: The calculated binding energy (in kcal/mol) of the weak bound CO ligand in the solvated VM(CO)_10_^+^ at the B3LYP, BLYP, PBE, and TPSS level with the def2-TZVP basis set; Table S5: The cartesian coordinates (Å) of the structures of VM(CO)_9_^+^ optimized at B3LYP/def2-TZVP level.
