# Supplementary material for: Infrared Photodissociation Spectroscopy of Dinuclear Vanadium-Group Metal Carbonyl Complexes: Diatomic Synergistic Activation of Carbon Monoxide
Source: Molecules. 2024 Jun 14;29(12):2831. doi: 10.3390/molecules29122831 (PMC11206424; doi:10.3390/molecules29122831)
Supplement: Supplementary file 1 [file molecules-29-02831-s001.zip › molecules-3031210-supplementary.pdf]

Supplementary Materials

Infrared Photodissociation Spectroscopy of Dinuclear Vanadium-group Metal Carbonyl Complexes: Diatomic Synergistic Activation of Carbon Monoxide

Jin Hu , Xuefeng Wang*

Shanghai Key Laboratory of Chemical Assessment and Sustainability, School of Chemical Science and Engineering, Tongji University, 1239 Siping Road, Shanghai 200092, China; hujin328@tongji.edu.cn

* Correspondence: xfwang@tongji.edu.cn

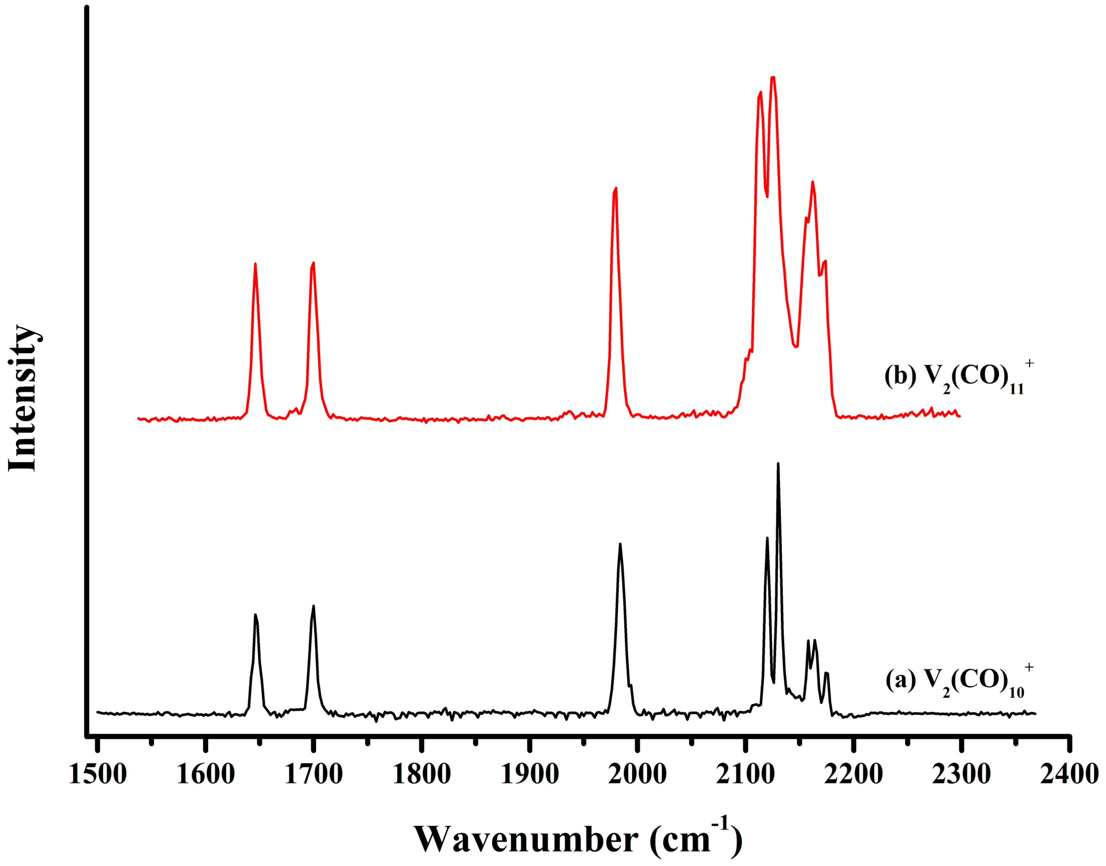

Figure S1. The comparison of experimental infrared photodissociation spectra of the $V_2(CO)_{10,11}^+$ cation complexes.

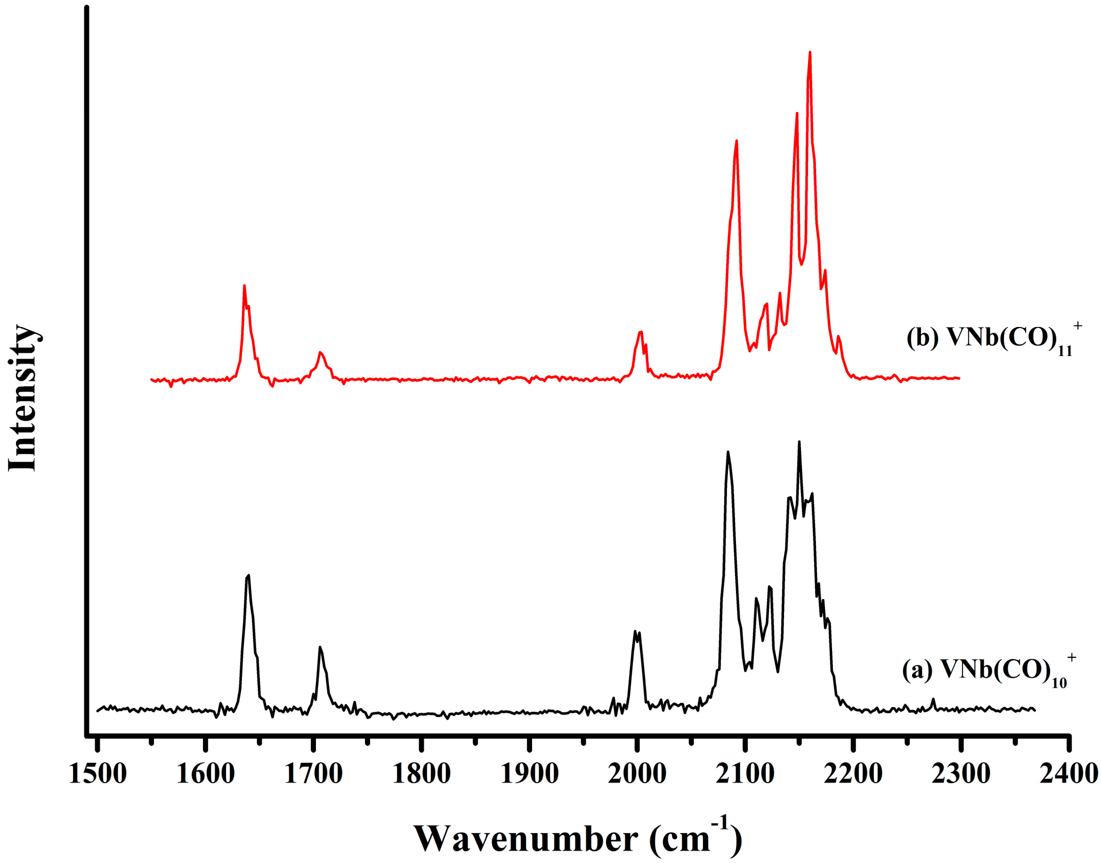

Figure S2. The comparison of experimental infrared photodissociation spectra of the $VNb(CO)_{10,11}^{+}$ cation complexes.

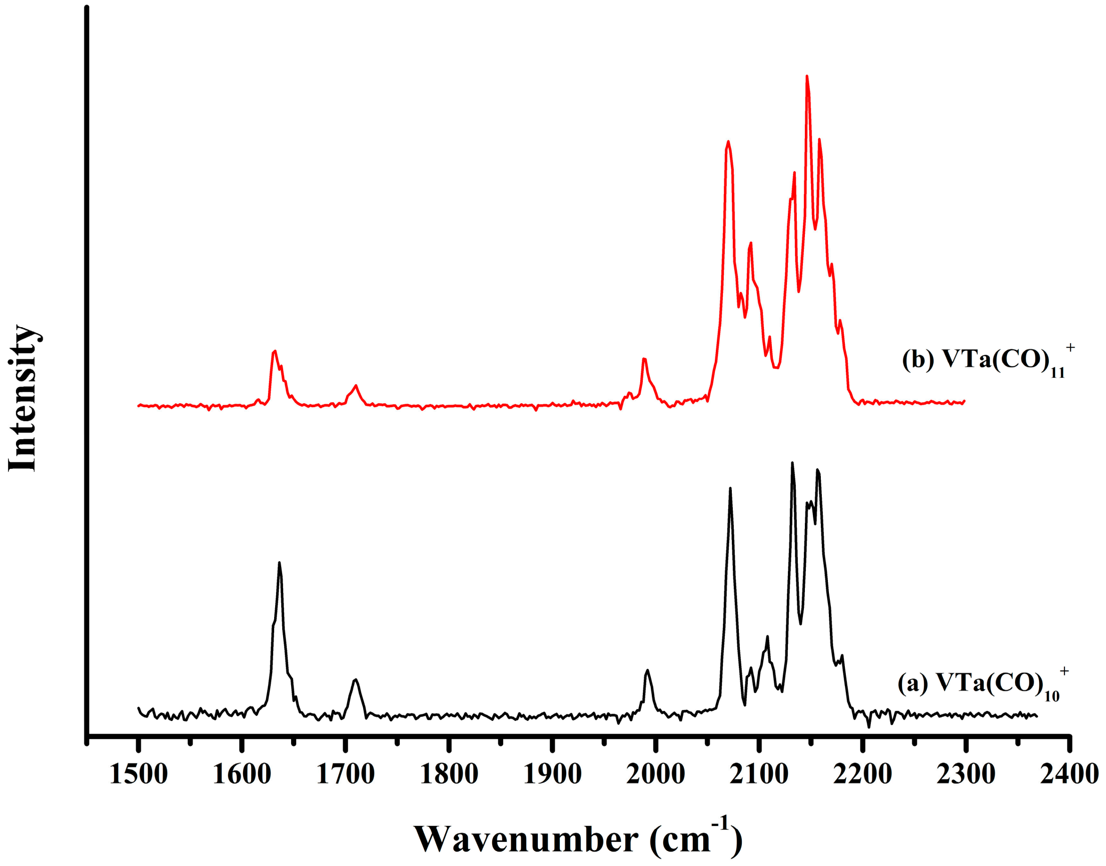

Figure S3. The comparison of experimental infrared photodissociation spectra of the $VTa(CO)_{10,11}^{+}$ cation complexes.

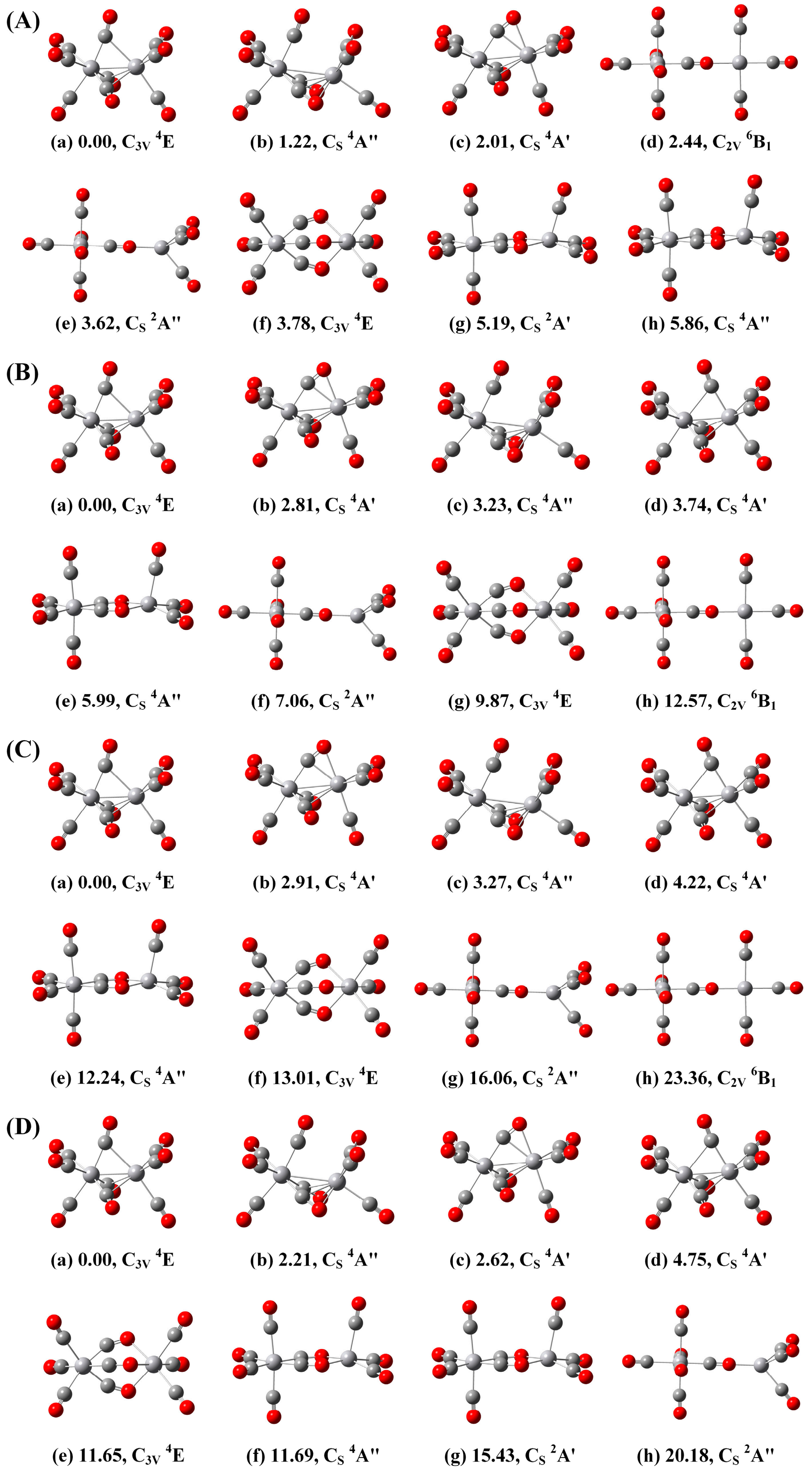

Figure S4. The calculated lowest-lying structures of the strongly bonded $V_2(CO)_9^+$ cation at the (A) B3LYP, (B) BLYP, (C) PBE, and (D) TPSS level with the def2-TZVP basis set. The symmetry, electronic state, and relative energy with the ZPE correction of each isomer (ΔE in kcal/mol) are indicated.

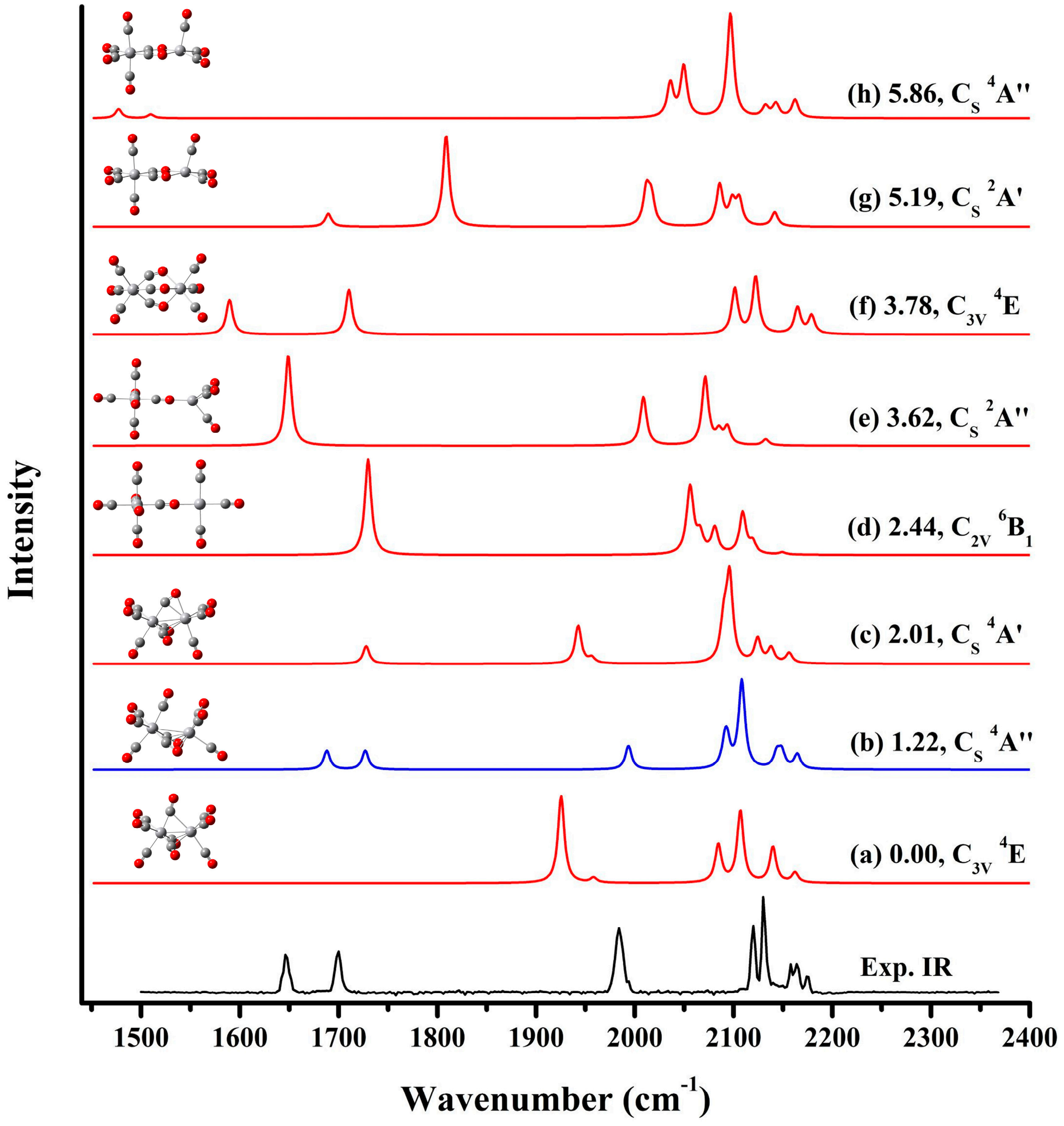

Figure S5. The experimental infrared photodissociation spectrum of $V_2(CO)_{10}^+$ and the simulated vibrational spectra of the eight lowest-lying $V_2(CO)_9^+$ cation complexes in the carbonyl stretching frequency region. The simulated spectrum that agrees well with the experimental spectrum is plotted in blue. The indicated symmetry, electronic state, and relative energy (ΔE in kcal/mol) with the ZPE correction of each isomer are derived from the B3LYP calculations.

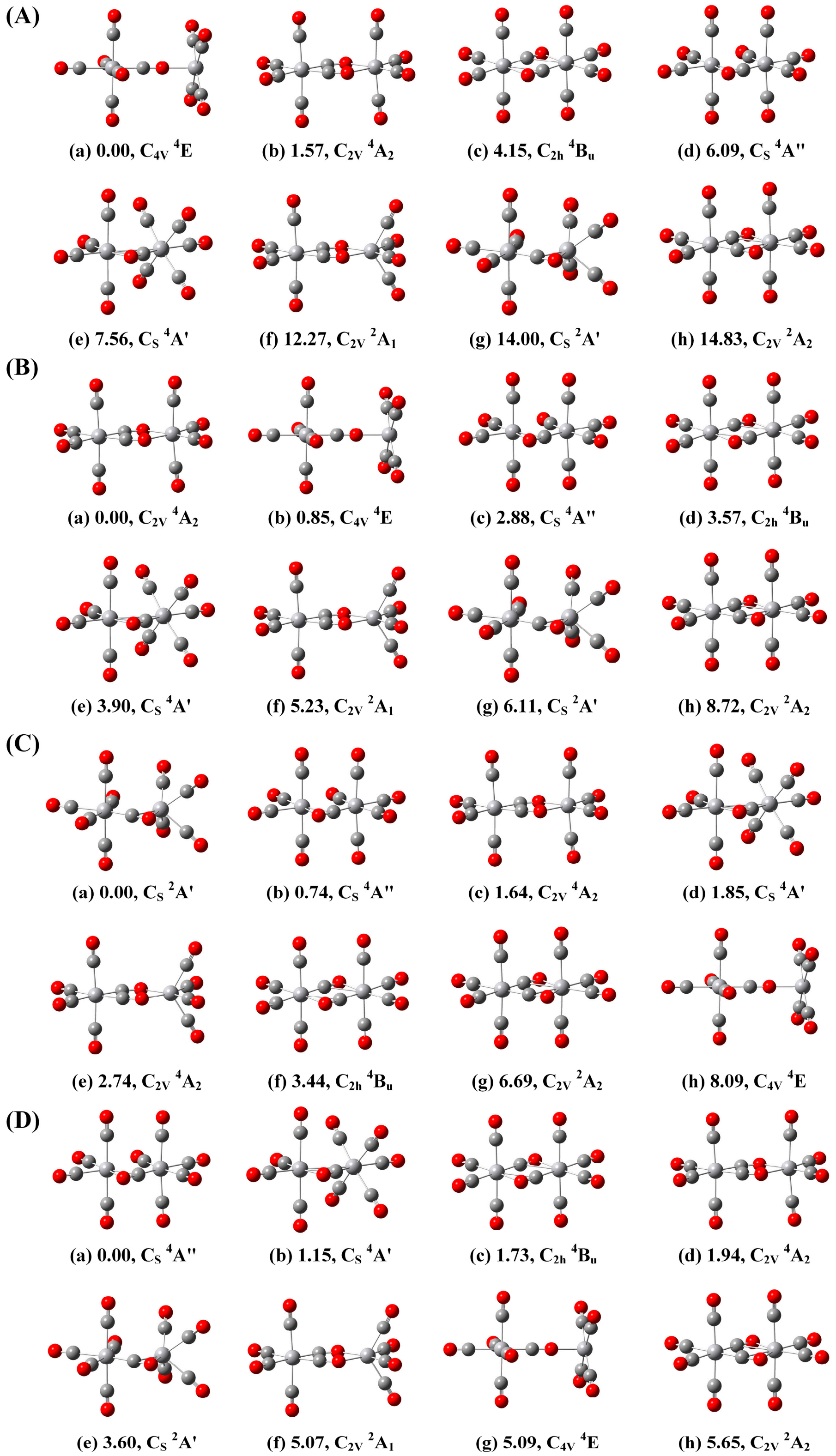

Figure S6. The calculated lowest-lying structures of the strongly bonded $V_2(CO)_{10}^+$ cation at the (A) B3LYP, (B) BLYP, (C) PBE, and (D) TPSS level with the def2-TZVP basis set. The symmetry, electronic state, and relative energy with the ZPE correction of each isomer (ΔE in kcal/mol) are indicated.

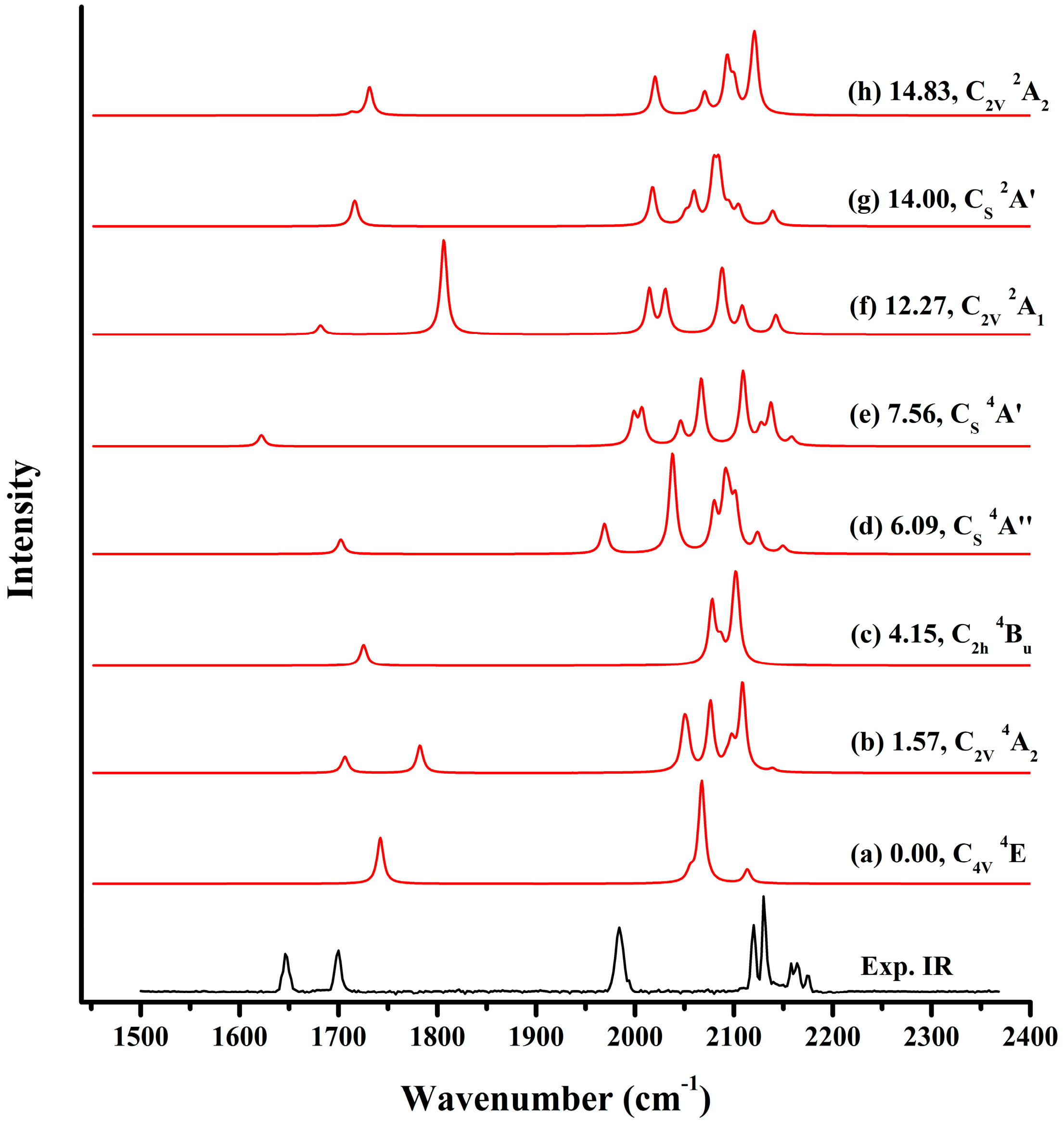

Figure S7. The experimental infrared spectrum of $V_2(CO)_{10}^+$ and the simulated vibrational spectra of the low-lying strongly bonded $V_2(CO)_{10}^+$ cation complexes in the carbonyl stretching frequency region. The indicated symmetry, electronic state, and relative energy (ΔE in kcal/mol) with the ZPE correction of each isomer are derived from the B3LYP calculations.

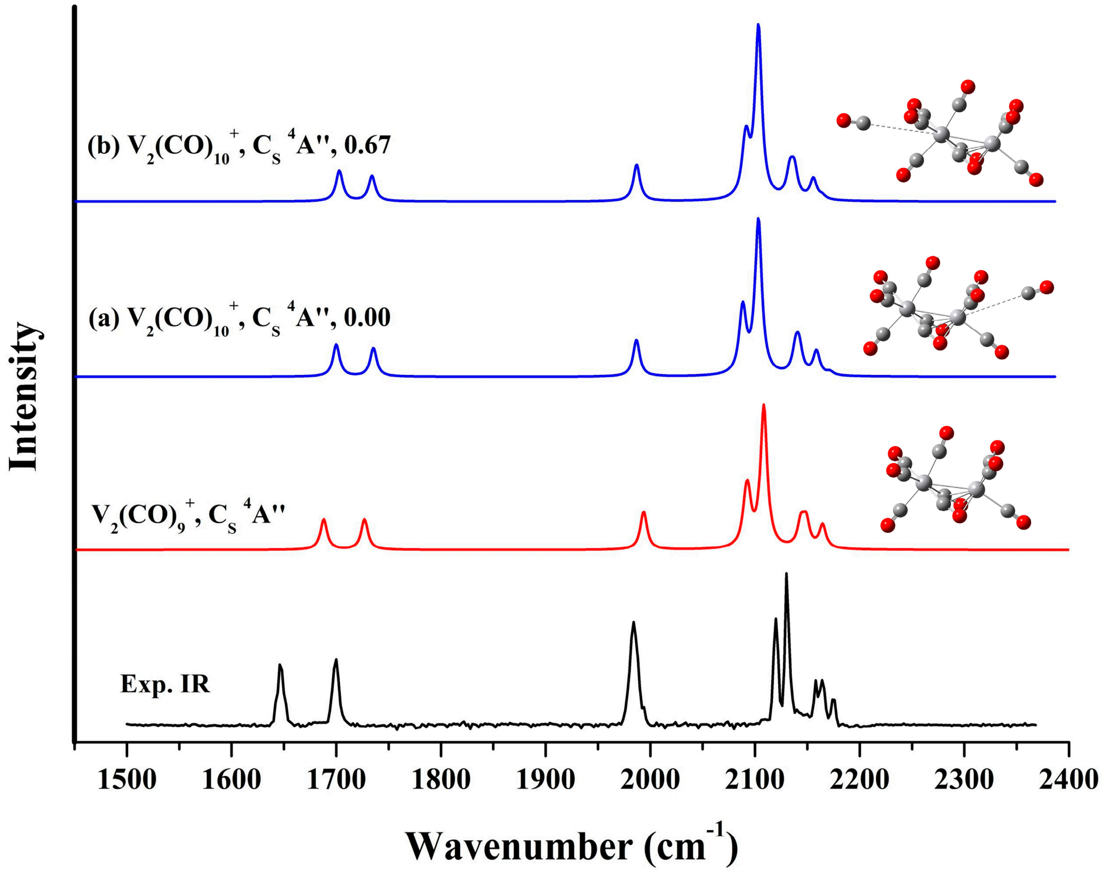

Figure S8. Comprisons of the experimental infrared spectrum of $V_2(CO)_{10}^+$ and the simulated vibrational spectra of the saturated $V_2(CO)_9^+$ cation and the low-lying CO-tagged $V_2(CO)_{10}^+$ complexes in the carbonyl stretching frequency region. The indicated symmetry, electronic state, and relative energy (ΔE in kcal/mol) with the ZPE correction of each structure are derived from the B3LYP calculations.

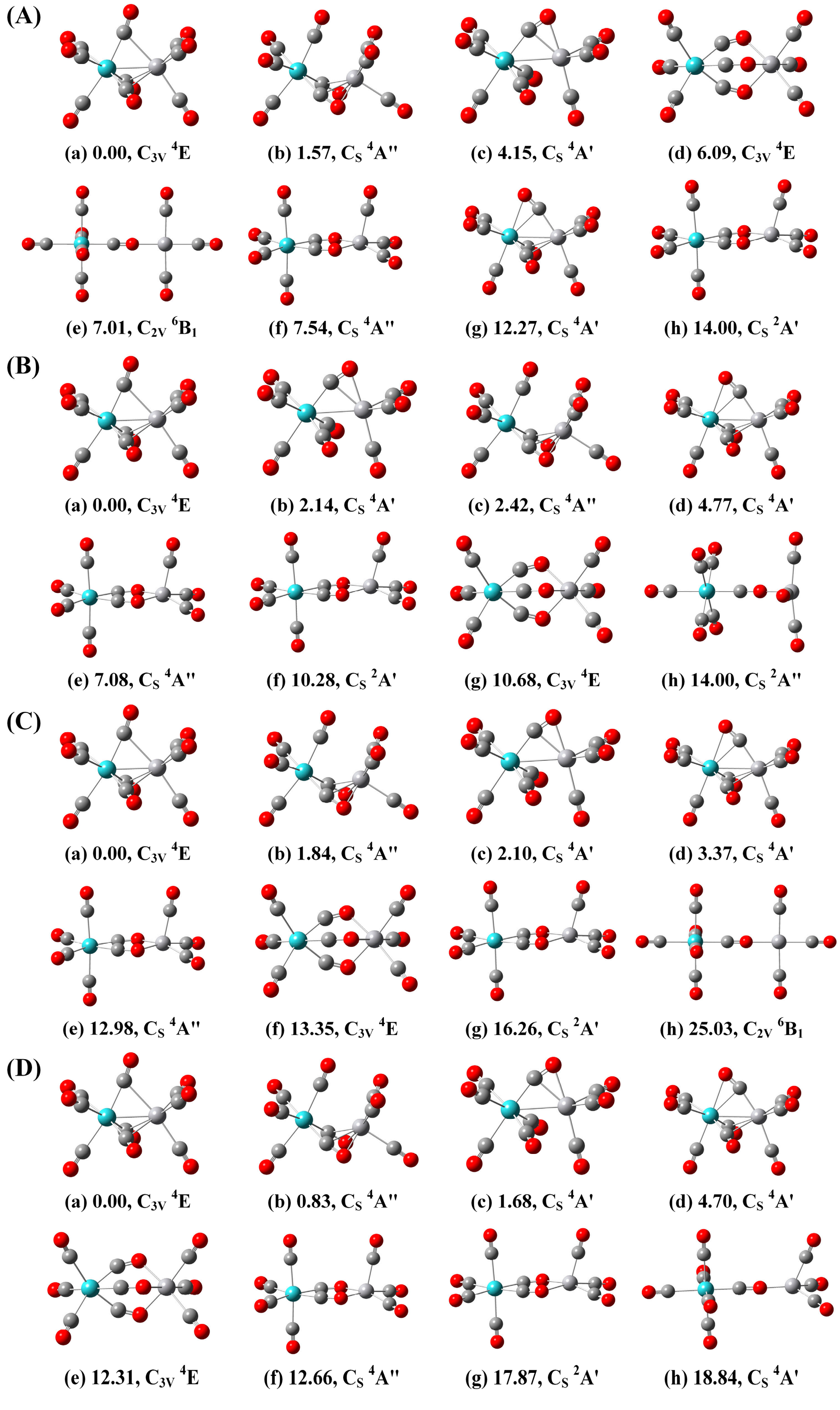

Figure S9. The calculated lowest-lying structures of the strongly bonded $VNb(CO)_9^+$ cation at the (A) B3LYP, (B) BLYP, (C) PBE, and (D) TPSS level with the def2-TZVP basis set. The symmetry, electronic state, and relative energy with the ZPE correction of each isomer (ΔE in kcal/mol) are indicated.

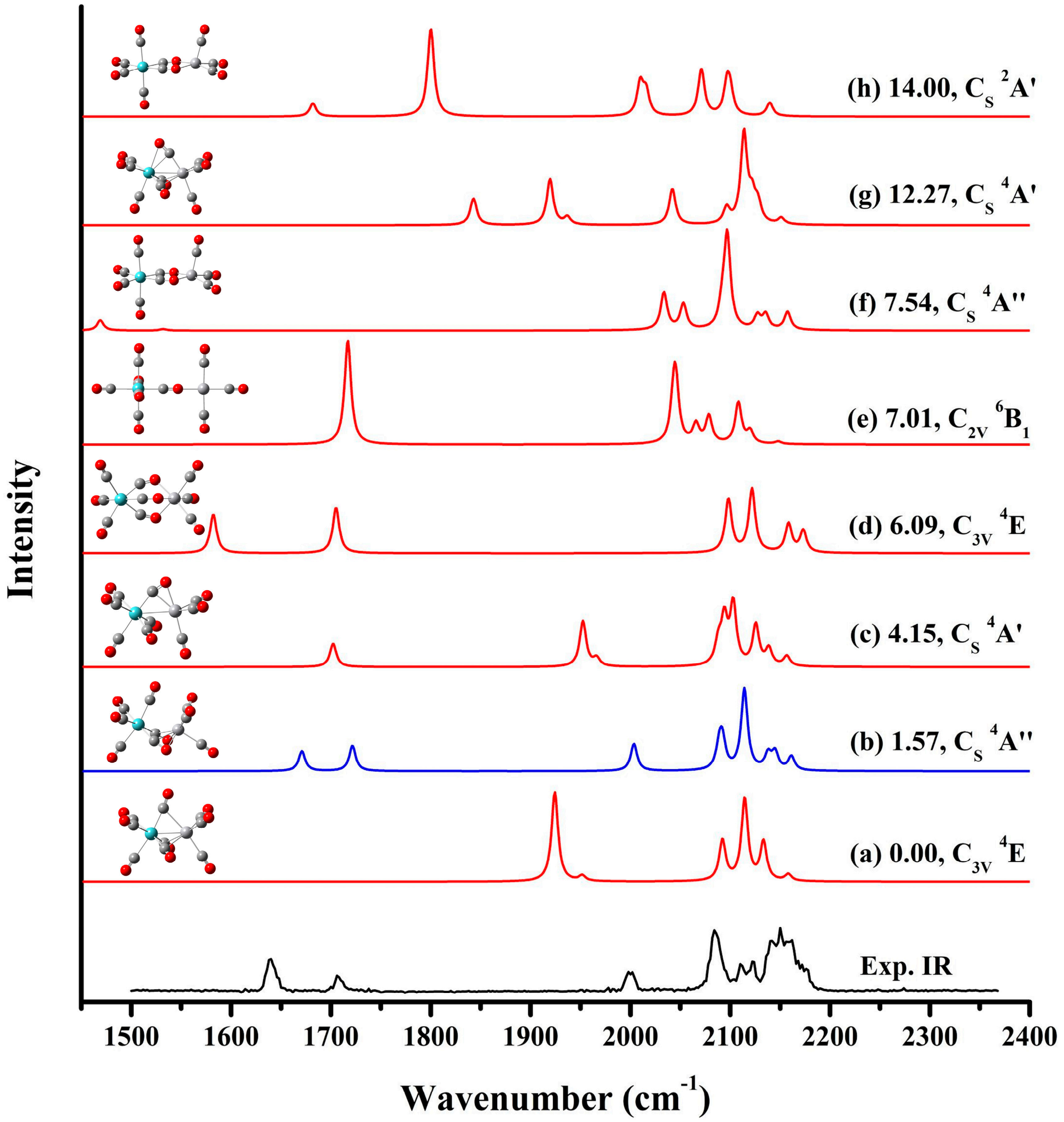

Figure S10. The experimental infrared photodissociation spectrum of $VNb(CO)_{10}^{+}$ and the simulated vibrational spectra of the eight lowest-lying $VNb(CO)_{9}^{+}$ cation complexes in the carbonyl stretching frequency region. The simulated spectrum that agrees well with the experimental spectrum is plotted in blue. The indicated symmetry, electronic state, and relative energy (ΔE in kcal/mol) with the ZPE correction of each isomer are derived from the B3LYP calculations.

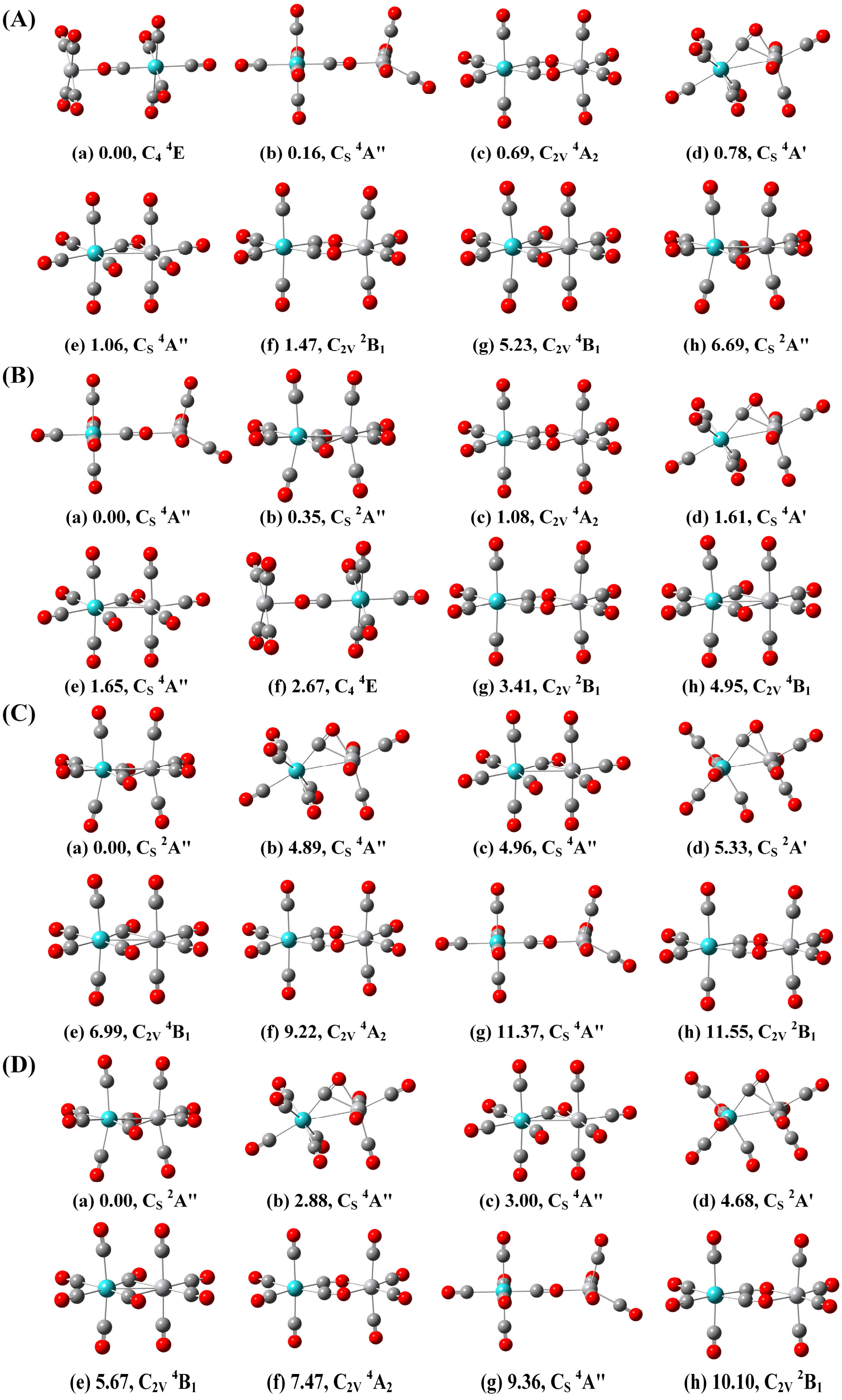

Figure S11. The calculated lowest-lying structures of the strongly bonded $VNb(CO)_{10}^+$ cation at the (A) B3LYP, (B) BLYP, (C) PBE, and (D) TPSS level with the def2-TZVP basis set. The symmetry, electronic state, and relative energy with the ZPE correction of each isomer (ΔE in kcal/mol) are indicated.

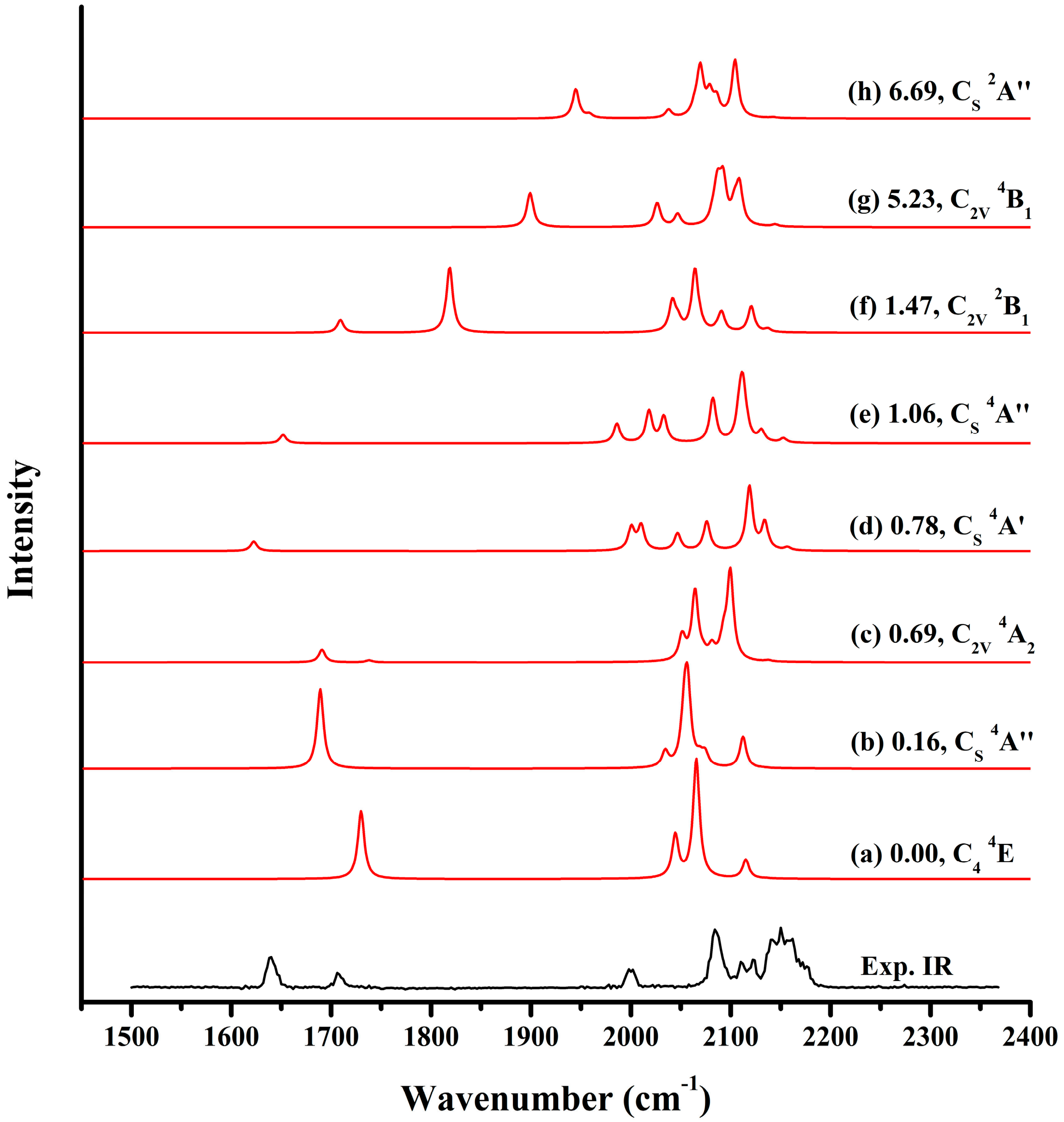

Figure S12. The experimental infrared spectrum of $V_2(CO)_{10}^+$ and the simulated vibrational spectra of the low-lying strongly bonded $V_2(CO)_{10}^+$ cation complexes in the carbonyl stretching frequency region. The indicated symmetry, electronic state, and relative energy (ΔE in kcal/mol) with the ZPE correction of each isomer are derived from the B3LYP calculations.

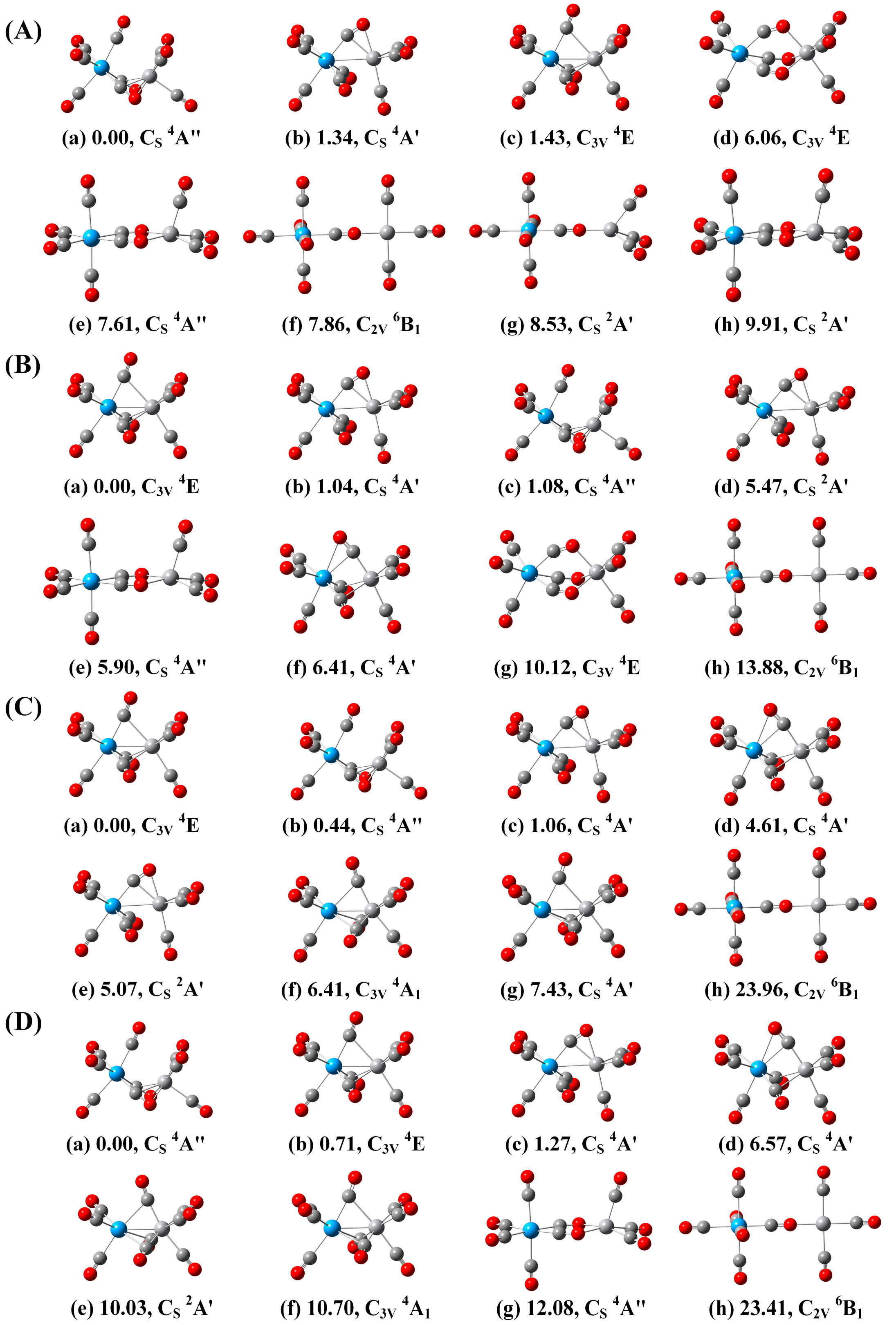

Figure S13. The calculated lowest-lying structures of the strongly bonded $VTa(CO)_9^+$ cation at the (A) B3LYP, (B) BLYP, (C) PBE, and (D) TPSS level with the def2-TZVP basis set. The symmetry, electronic state, and relative energy with the ZPE correction of each isomer (ΔE in kcal/mol) are indicated.

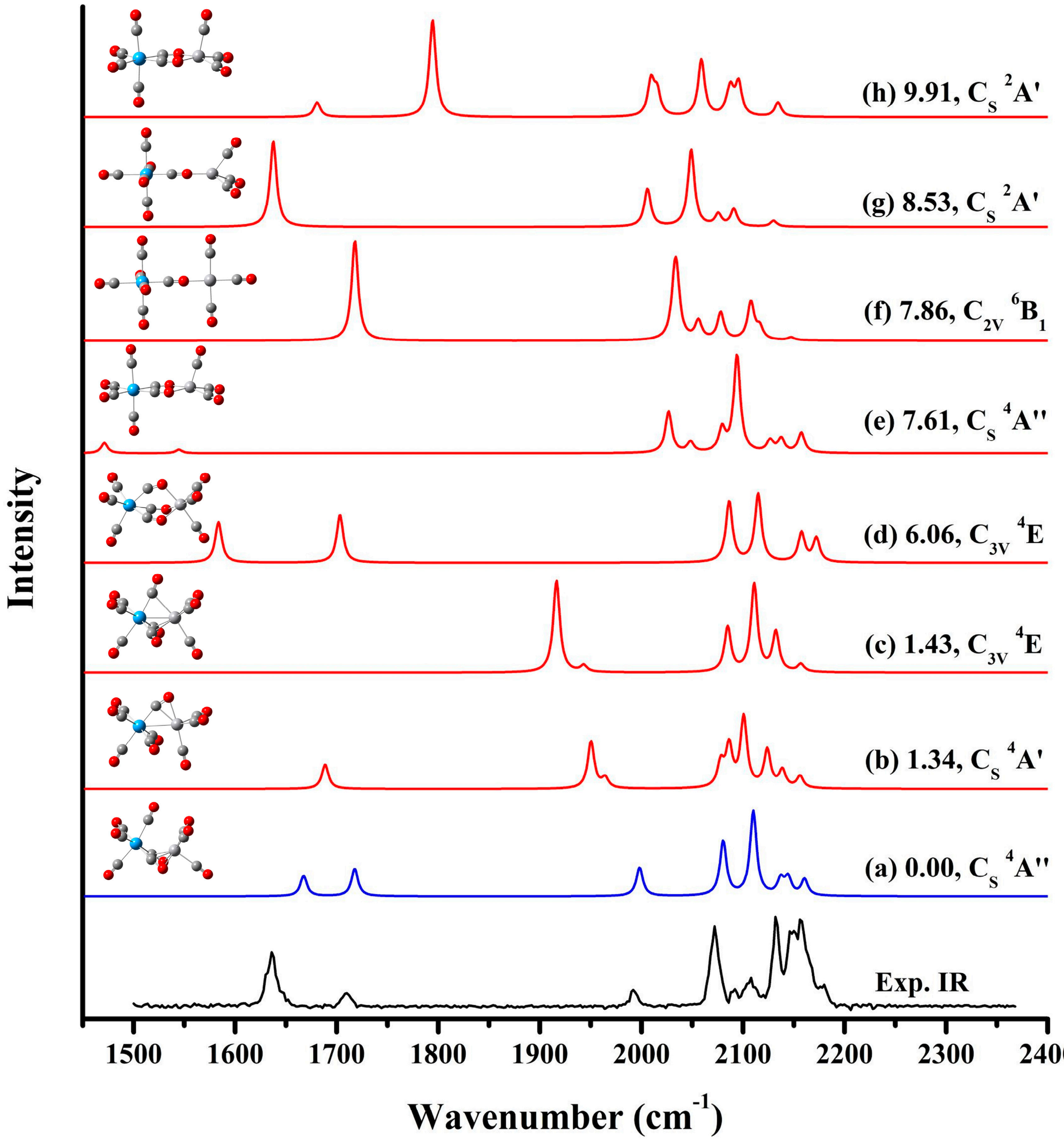

Figure S14. The experimental infrared photodissociation spectrum of $VTa(CO)_{10}^{+}$ and the simulated vibrational spectra of the eight lowest-lying $VTa(CO)_9^{+}$ cation complexes in the carbonyl stretching frequency region. The simulated spectrum that agrees well with the experimental spectrum is plotted in blue. The indicated symmetry, electronic state, and relative energy with the ZPE correction of each isomer (ΔE in kcal/mol) are derived from the B3LYP calculations.

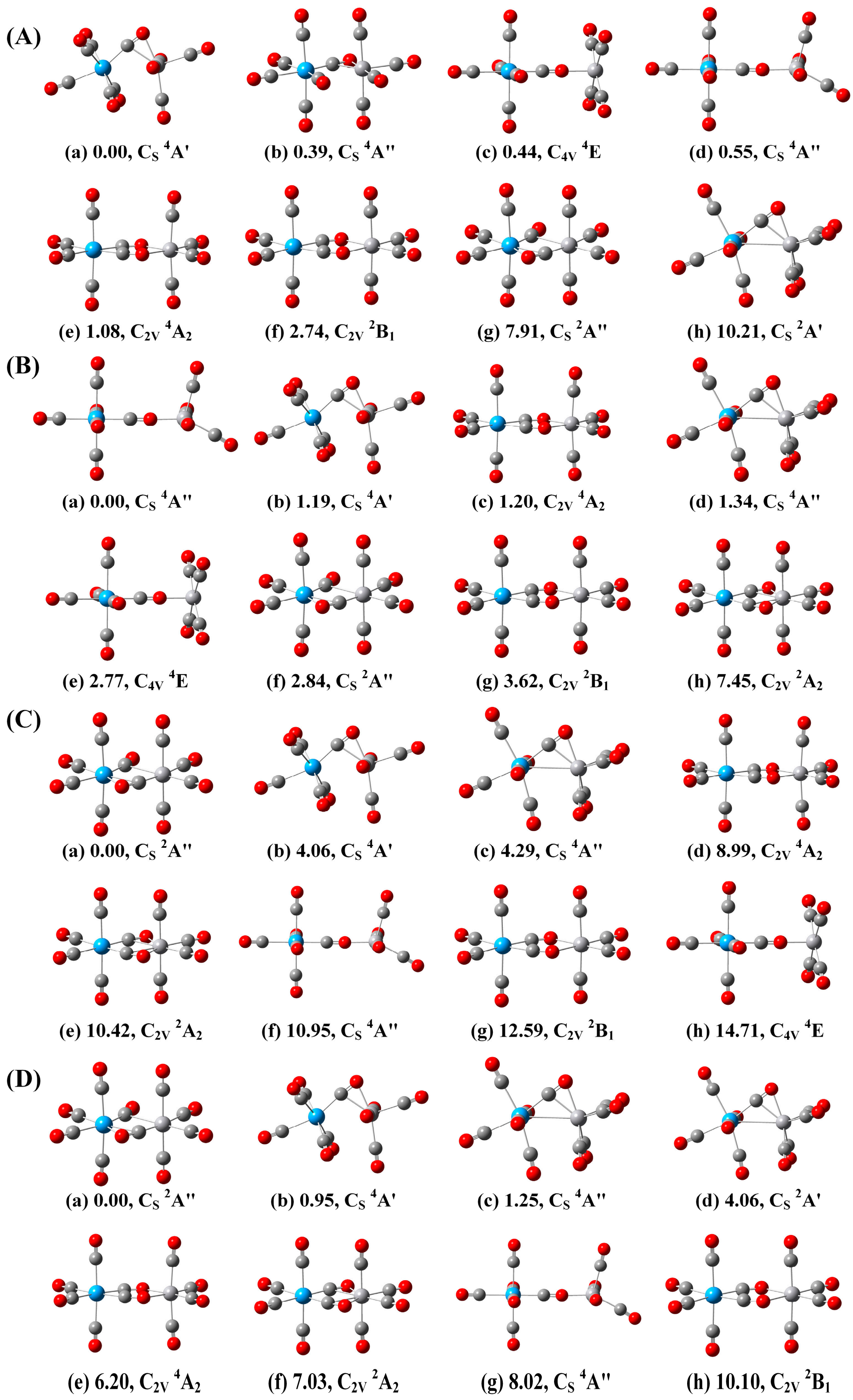

Figure S15. The calculated lowest-lying structures of the strongly bonded $VNb(CO)_{10}^+$ cation at the (A) B3LYP, (B) BLYP, (C) PBE, and (D) TPSS level with the def2-TZVP basis set. The symmetry, electronic state, and relative energy with the ZPE correction of each isomer (ΔE in kcal/mol) are indicated.

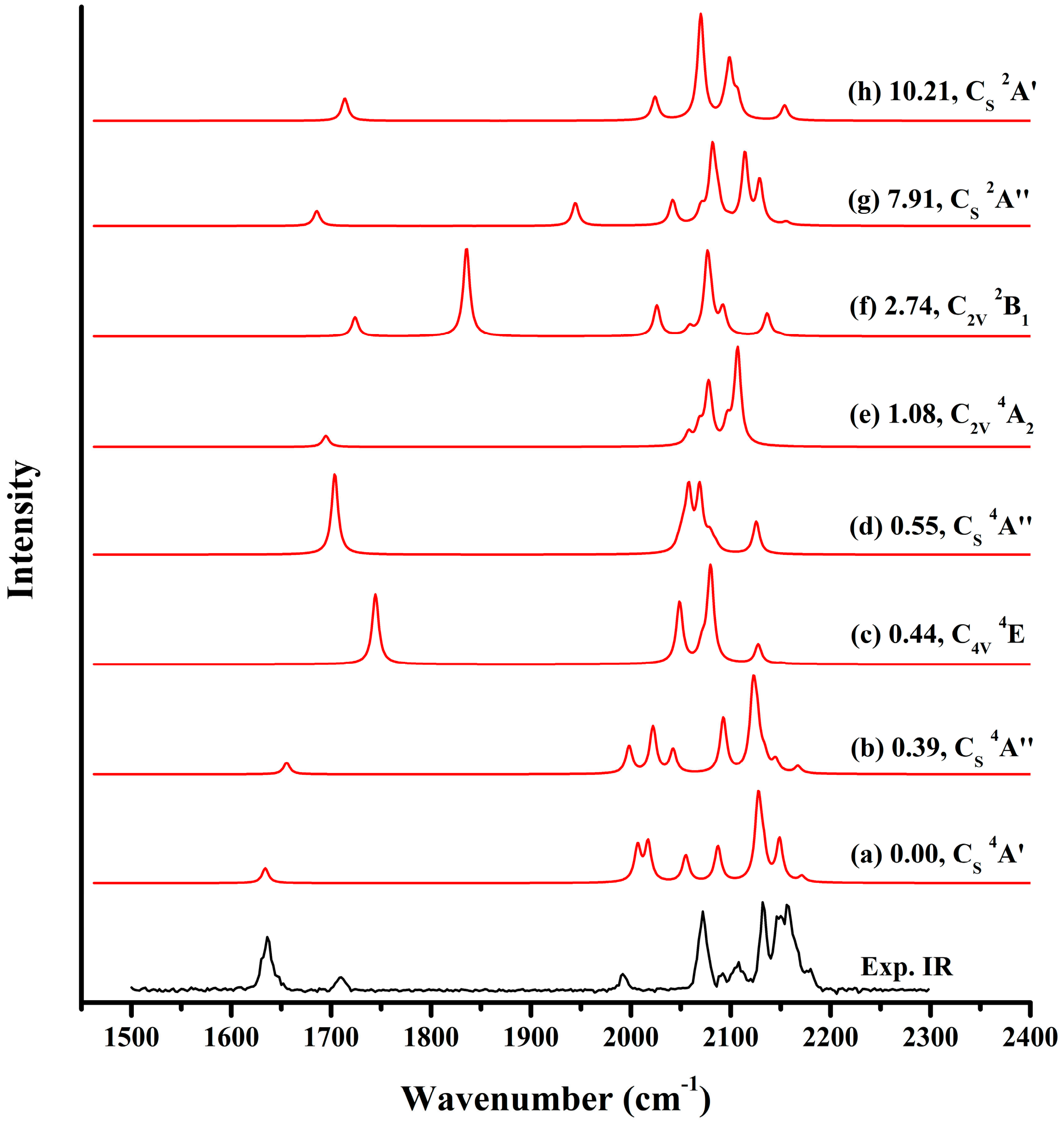

Figure S16. The experimental infrared spectrum of $VTa(CO)_{10}^{+}$ and the simulated vibrational spectra of the low-lying strongly bonded $VTa(CO)_{10}^{+}$ cation complexes in the carbonyl stretching frequency region. The indicated symmetry, electronic state, and relative energy with the ZPE correction of each isomer (ΔE in kcal/mol) are derived from the B3LYP calculations.

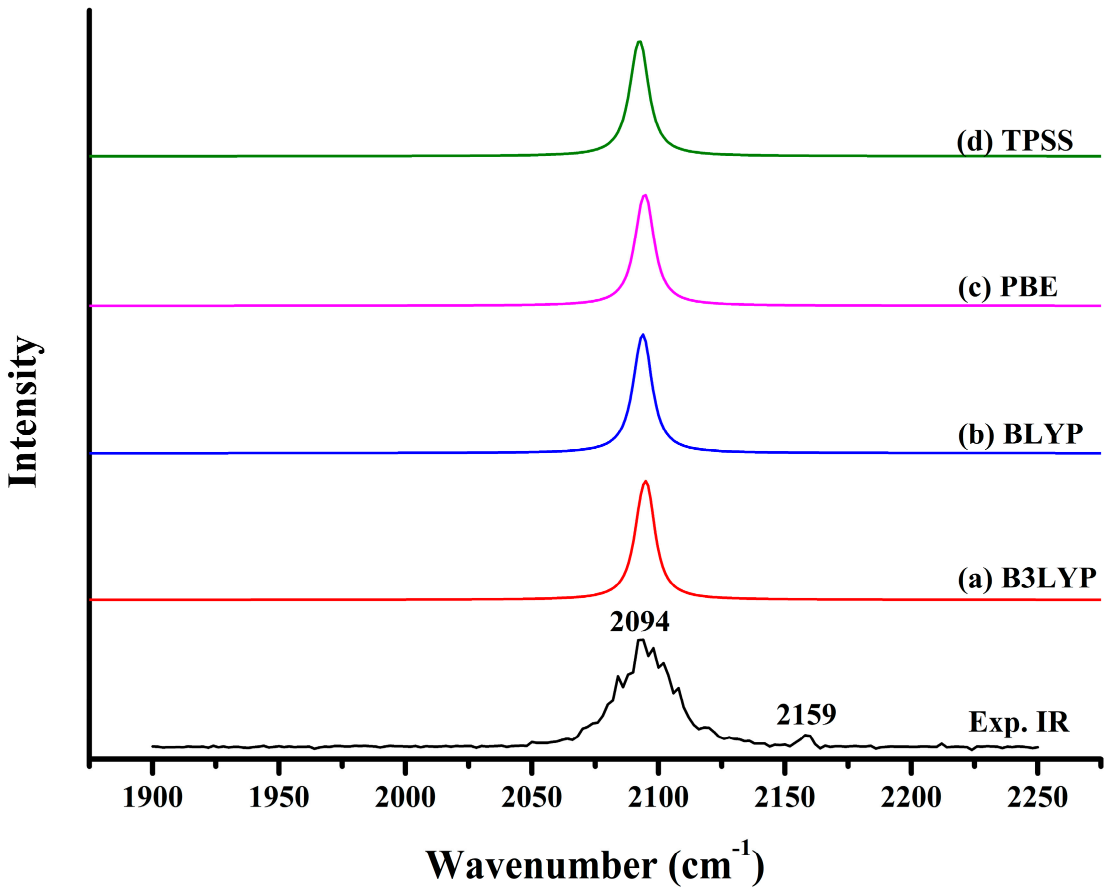

Figure S17. The comprison between the experimental vibrational spectrum of the solvated $V(CO)_7^+$ cluster cation and the simulated spectra of saturated $V(CO)_6^+$ cation derived from calculations at (a) B3LYP, (b) BLYP, (c) PBE, and (d) TPSSh level in conjunction with the def2-TZVP basis set. The simulated spectra were obtained from scaled harmonic frequencies with a scaled factor of 0.968 for B3LYP, 1.015 for BLYP, 1.008 for PBE, and 1.000 for TPSS, respectively.

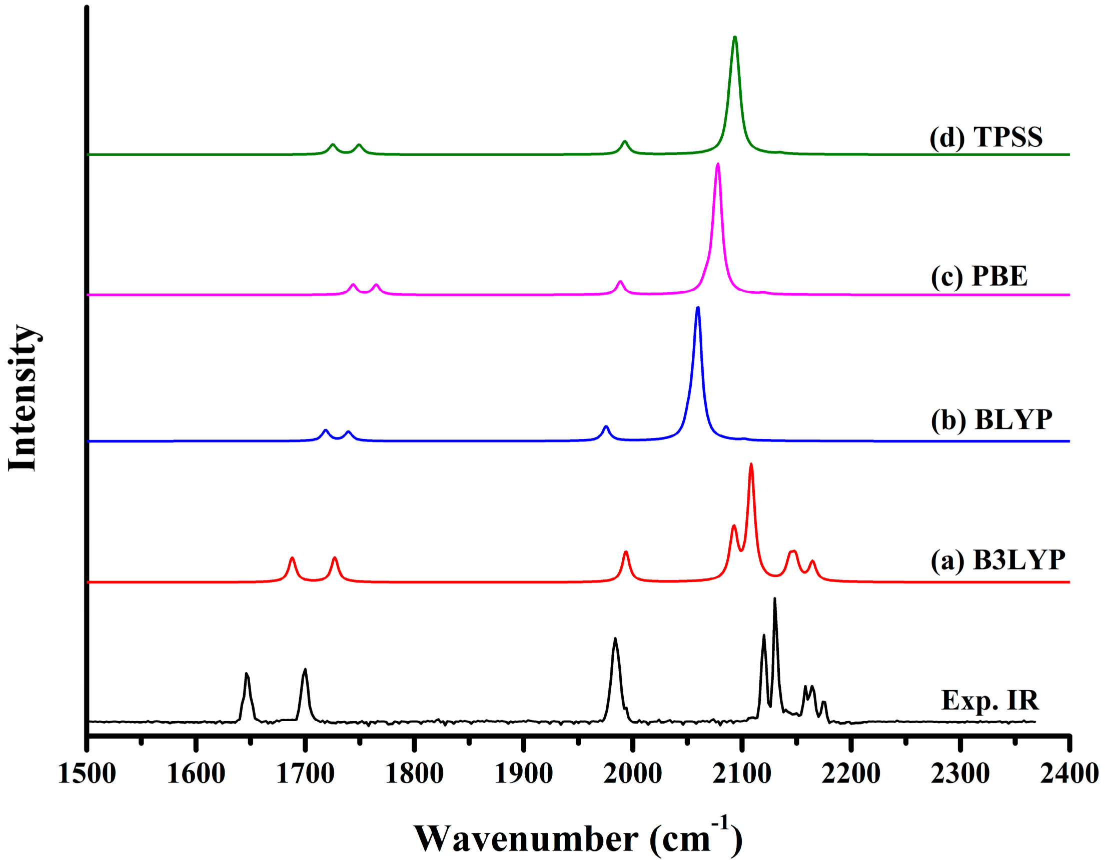

Figure S18. The comprison between the experimental vibrational spectrum of the solvated $V_2(CO)_{10}^+$ cluster cation and the simulated spectra of saturated $V_2(CO)_9^+$ cation derived from calculations at (a) B3LYP, (b) BLYP, (c) PBE, and (d) TPSSh level in conjunction with the def2-TZVP basis set. The simulated spectra were obtained from scaled harmonic frequencies with a scaled factor of 0.968 for B3LYP, 1.015 for BLYP, 1.008 for PBE, and 1.000 for TPSS, respectively.

Table S1. The calculated charge and spin population of $VM(CO)_9^+$ based on natural population analysis.

Complex	Site	NPA charge	Spin population
$V_2(CO)_9^+$	V1	-2.583	0.405
(Cs ^{4}A")	V2	-0.410	2.564
	Side-on bridging CO	0.367	-0.061
	End-on semibridging CO	0.435	0.000
$VNb(CO)_9^+$	Nb1	-1.653	0.260
(Cs ^{4}A")	V2	-0.431	2.590
	Side-on bridging CO	0.174	-0.030
	End-on semibridging CO	0.300	0.015
$VTa(CO)_9^+$	Ta1	-1.367	0.228
(Cs ^{4}A")	V2	-0.439	2.592
	Side-on bridging CO	0.116	-0.028
	End-on semibridging CO	0.256	0.018

Table S2. Orbital composition of the AdNDP orbitals of the quartet $VNb(CO)_9^+$ complex cations based on natural atomic orbital method.

Orbital	Composition
1# 1c-1e	V [$3d_{xy}$(11%) + $3d_{x2-y2}$(18%) + $3d_{z2}$(70%)]
2# 1c-1e	V [$3d_{xz}$(22%) + $3d_{yz}$(78%)]
3# 1c-1e	V [$3d_{xy}$(51%) + $3d_{x2-y2}$(49%)]
1# 2c-2e	Nb [5s(8%) + 5p(10%) + 4d(14%)] + CO [5σ(68%)]
2# 2c-2e	Nb [5s(8%) + 5p(10%) + 4d(14%)] + CO [5σ(68%)]
1# 3c-2e	V [4s(3%) + 4p(5%) + 3d(7%)] + CO [2π(85%)]
2# 3c-2e	V [4s(3%) + 4p(5%) + 3d(7%)] + CO [2π(85%)]
6c-2e	V [$3d_{xy}$(4%) + $3d_{x2-y2}$(5%) + $3d_{z2}$(6%)] + Nb [$4d_{xy}$(15%) + $4d_{x2-y2}$(18%) + $4d_{z2}$(24%)] + 2 × CO [2π*(13%)]

Table S3. The calculated energy (in a.u.) of quartet V_2^+ dimer, quintet V^+ cation ($3d^4$), and quartet V atom ($4s^2 3d^3$), as well as the bond dissociation energy (BDE, in eV) at the B3LYP, BLYP, PBE, and TPSS level with the def2-TZVP basis set.

Complex	$E(V_2^+)$	$E(V^+)$	E(V)	BDE(eV)
B3LYP	-1887.656	-943.673	-943.912	1.947
BLYP	-1887.760	-943.683	-943.922	4.225
PBE	-1887.233	-943.418	-943.649	4.500
TPSS	-1887.746	-943.687	-943.914	3.970

Table S4. The calculated binding energy (in kcal/mol) of the weak bound CO ligand in the solvated $VM(CO)_{10}^+$ at the B3LYP, BLYP, PBE, and TPSS level with the def2-TZVP basis set.

Complex	$V_2(CO)_{10}^+$	$VNb(CO)_{10}^+$	$VTa(CO)_{10}^+$
B3LYP	1.35	1.32	1.27
BLYP	0.93	0.90	0.87
PBE	1.99	1.95	1.91
TPSS	1.59	1.55	1.53

Table S5. The cartesian coordinates (Å) of the structures of $VM(CO)_9^+$ optimized at B3LYP/def2-TZVP level.

Complex	Atom	x	y	z
$V_2(CO)_9^+$	V	0.090631	-1.44555	0
	V	0.239041	1.541899	0
	C	0.964178	-0.25077	1.211919
	O	1.518083	0.710571	1.631678
	C	0.964178	-0.25077	-1.21192
	O	1.518083	0.710571	-1.63168
	C	-1.77405	-0.70953	0
	O	-2.86546	-0.37826	0
	C	1.754377	-2.6591	0
	O	2.685904	-3.29579	0
	C	-0.62622	-2.60914	1.572751
	O	-0.99112	-3.1969	2.463271
	C	-0.62622	-2.60914	-1.57275
	O	-0.99112	-3.1969	-2.46327
	C	1.32576	3.382924	0
	O	1.930362	4.326875	0
	C	-1.09806	2.184887	1.543706
	O	-1.79597	2.522325	2.353772
	C	-1.09806	2.184887	-1.54371
	O	-1.79597	2.522325	-2.35377
$VNb(CO)_9^+$	Nb	0.076061	-1.35842	0
(Cs, $^4A''$)	V	0.256034	1.750015	0
	C	0.998328	-0.02477	1.263436
	O	1.550798	0.962496	1.626196
	C	0.998328	-0.02477	-1.26344
	O	1.550798	0.962496	-1.6262
	C	-1.94661	-0.59633	0
	O	-3.0378	-0.27015	0
	C	1.926094	-2.59099	0
	O	2.876588	-3.19842	0
	C	-0.67247	-2.57239	1.749868
	O	-1.0168	-3.11869	2.673851
	C	-0.67247	-2.57239	-1.74987
	O	-1.0168	-3.11869	-2.67385
	C	-1.1135	2.323216	1.537951
	O	-1.83454	2.607216	2.34915
	C	1.287567	3.62228	0
	O	1.867548	4.58184	0
	C	-1.1135	2.323216	-1.53795
	O	-1.83454	2.607216	-2.34915
$VTa(CO)_9^+$	Ta	0.058183	-1.17258	0

(Cs, $^4A''$)	V	0.266471	1.948074	0
	C	0.992421	0.164451	1.263105
	O	1.545323	1.1534	1.626118
	C	0.992421	0.164451	-1.2631
	O	1.545323	1.1534	-1.62612
	C	-1.96178	-0.41125	0
	O	-3.0523	-0.0792	0
	C	1.872232	-2.44511	0
	O	2.80248	-3.08442	0
	C	-0.69024	-2.3492	1.755842
	O	-1.03794	-2.88151	2.688263
	C	-0.69024	-2.3492	-1.75584
	O	-1.03794	-2.88151	-2.68826
	C	1.30295	3.818978	0
	O	1.880592	4.779944	0
	C	-1.10362	2.536109	1.531134
	O	-1.82524	2.844991	2.332784
	C	-1.10362	2.536109	-1.53113
	O	-1.82524	2.844991	-2.33278
